# RAB4A is a master regulator of cancer cell stemness upstream of NUMB–NOTCH signaling

**DOI:** 10.1038/s41419-024-07172-w

**Published:** 2024-10-27

**Authors:** Subbulakshmi Karthikeyan, Patrick J. Casey, Mei Wang

**Affiliations:** 1 Program in Cancer Stem Cell Biology, Duke-NUS Medical School, Singapore, Singapore; 2 Department of Pharmacology and Cancer Biology, Duke University Medical Center, Durham, NC USA; 3 Department of Biochemistry, National University of Singapore, Singapore, Singapore

**Keywords:** Cancer stem cells, Breast cancer, Self-renewal

## Abstract

Cancer stem cells (CSCs) are a group of specially programmed tumor cells that possess the characteristics of perpetual cell renewal, increased invasiveness, and often, drug resistance. Hence, eliminating CSCs is a major challenge for cancer treatment. Understanding the cellular programs that maintain CSCs, and identifying the critical regulators for such programs, are major undertakings in both basic and translational cancer research. Recently, we have reported that RAB4A is a major regulator of epithelial-to-mesenchymal transition (EMT) and it does so mainly through regulating the activation of RAC1 GTPase. In the current study, we have delineated a new signaling circuitry through which RAB4A transmits its control of cancer stemness. Using in vitro and in vivo studies, we show that RAB4A, as the upstream regulator, relays signal stepwise to NUMB, NOTCH1, RAC1, and then SOX2 to control the self-renewal property of multiple cancer cells of diverse tissue origins. Knockdown of NUMB, or overexpression of NICD (the active fragment NOTCH1) or SOX2, rescued the in vitro sphere-forming and in vivo tumor-forming abilities that were lost upon RAB4A knockdown. Furthermore, we discovered that the chain of control is mostly through transcriptional regulation at every step of the pathway. The discovery of the novel signaling axis of RAB4A–NUMB–NOTCH–SOX2 opens the path for further expansion of the signaling chain and for the identification of new regulators and interacting proteins important for CSC functions, which can be explored to develop new and effective therapies.

## Introduction

Cancer stem cells (CSCs) possess a self-renewal capacity that is important not only for tumor initiation, maintenance, and progression, but also enhanced resistance to many modalities of therapies, contributing to treatment failure and cancer recurrence [1, 2]. Epithelial–mesenchymal-transition (EMT), a biological process where epithelial cells acquire mesenchymal-like characteristics, is associated with the acquisition of stem cell-like properties and tumor progression [3, 4]. Due to their central roles in cancer, targeting EMT and cancer stemness has emerged as a promising strategy to improve cancer treatment outcomes [5]. To this end, identifying the factors that are important for EMT and cancer stemness can pave the way for the development of targeted therapies aimed at disrupting EMT and eliminating CSCs to improve clinical outcomes.

NUMB is a protein that plays a crucial role in the regulation of cell fate, including its involvement in CSCs. During development, NUMB is a determinant in the asymmetric division of stem cells to maintain tissue homeostasis; in the cancer cell, it acts mostly as a tumor suppressor in reducing self-renewal capacity and stemness thus limiting tumor growth [6, 7]. Though a known tumor suppressor, NUMB has context-dependent roles in specific cancers highlighting the complexity of its functions and emphasizing the importance of a clear understanding of the signaling circuitry [8–12].

NOTCH protein signaling is also highly conserved in various cellular processes, including development, differentiation, and tissue homeostasis. In recent years, the pro-cancer roles of NOTCH signaling in cancer stemness and drug resistance have garnered significant attention [13–15]. NOTCH signaling cross-talks with several other pathways involved in drug resistance, such as PI3K/Akt and MAPK/ERK. As such, therapeutic targeting of NOTCH and its downstream effectors has shown promise in preclinical and clinical settings [16]. As with many other proteins that play roles in cancer, the mechanism of regulation by NOTCH is also cell context-dependent [17–21].

SOX2 (sex-determining region Y-box 2) is one of the key transcription factors involved in maintaining the pluripotency and self-renewal capacity of embryonic stem cells; it is one of the four so-called Yamanaka factors that induce pluripotent stem cells [22]. In cancer, SOX2 expression has been detected in CSC populations across various tumor types [23, 24]. It interacts with other transcription factors and signaling pathways to orchestrate the balance between self-renewal and differentiation of CSCs. As such, SOX2 is known to induce EMT and mediate drug resistance [25–28]. Therefore, targeting SOX2 can potentially reduce cancer cell self-renewal capacity and enhance the effectiveness of cancer treatments; however, little progress has been made due to the difficulty in directly targeting transcription factors [27].

A common theme has emerged that the roles of the aforementioned cancer stem cell regulators are complex and cell context-dependent in biology and in cancer, which underscores the importance of a better understanding of the regulatory mechanisms. Recently, we reported the RAB4A–RAC1 axis of signaling as a major regulator for EMT and cancer stemness [29]. RAC1, a member of the Rho family of small GTPases, is known for its roles in regulating cellular processes such as cytoskeletal dynamics, cell migration, and gene expression [30, 31]. Activation of RAC1 is also reported to contribute to the evasion of cell death induced by chemotherapy or targeted therapies, leading to resistance and tumor recurrence [32–39]. In addition, the crosstalk of RAC1 with many signaling pathways, such as those of Wnt/β-catenin, PI3K, and MAPK, is important in tumorigenesis [34, 35]. In the previous study, we found RAB4A to be a major upstream regulator of RAC1 activation on EMT and cancer stemness [29]. However, there are gaps/missing signaling components in the transmission of this regulation that need to be identified. In this paper, we report a novel signaling cascade of RAB4A–NUMB–NOTCH–RAC1–Sox2 as a major and fundamental driver in promoting cancer stemness and tumorigenesis, significantly extending the mechanistic understanding of the role of RAB4A–RAC1 regulation of EMT and stemness signaling.

## Materials and methods

### Cell culture

Human breast cancer cell lines MDA-MB-231, MCF7, human prostate cancer cells PC3, and human glioblastoma cells SNB19 were obtained from the American Type Culture Collection (ATCC) (Rockville, MD) and were tested mycoplasma negative. These cells were cultured in Dulbecco’s minimal essential medium (DMEM, Nacalai, California, USA) supplemented with 10% *v*/*v* FBS and penicillin (100 U/mL)/streptomycin (100 μg/mL) from Hyclone (IL, USA) following ATCC standard conditions. The actinomycin D (ThermoFisher Scientific, USA) treatment of cells was conducted using the established protocols [40].

### Generation of stable cell lines and transient transfection

HEK293T cells were used to make the viruses for stable knockdown and stable protein over-expression. Transfection was done by Lipofectamine 2000 (Invitrogen, Carlsbad, CA, USA) using standard lab protocols [41]. For transduction in the cell line of interest, the virus-containing-media from HEK293T cells was mixed with fresh 10% FBS DMEM at 50% *v*/*v* and with a final concentration of 8 μg/mL polybrene (#H9268, Sigma-Aldrich). The stable knockdown of RAB4A and NUMB was achieved using the shRNA plasmids obtained from Sigma-Aldrich, USA (Supplementary Table S1A). RAB4A overexpressing cells were generated as described previously [42]. NICD and SOX2 genes were amplified from the cDNA obtained from MDA-MB-231 cells (Supplementary Table S1B). RAC1 expressing constructs were generated as described previously [29]. RAB5A siRNA (#EHU053901, Sigma-Aldrich, USA) transient transfections were performed using Lipofectamine 3000 reagent (ThermoFisher Scientific, USA) and associated manufacturer’s protocol.

### Quantitative PCR (qPCR)

Total RNA extraction and cDNA conversion were done using Tissue Total RNA Mini Kit (FATRK 001-2, Favorgen Biotech Corporation) and ReverTra Ace qPCR RT Master Mix (FSQ-201, Toyobo). All qPCR measurements were performed using the BIO-RAD CFX96 (California, USA) and SYBR green (#4913850001, Roche, WI). The primers used for qPCR are listed in Supplementary Table S1C. Cancer stemness gene expression analysis was done according to the manufacturer’s protocol using the predesigned qPCR multiplex array “Cancer stem cells (SAB target list) H96, Bio-Rad, USA”. The heat map was generated using online Morpheus analysis software.

### Cellular fractionation, RAC1 pull-down assay, and immunoblotting

Nuclear extraction was performed using the protocol from Thermofisher for cellular fractionation (https://www.thermofisher.com/sg/en/home/references/protocols/cell-and-tissue-analysis/elisa-protocol/elisa-sample-preparation-protocols/nuclear-extraction-method-.html). Briefly, 5 × 10^6^ cells were collected in ice-cold PBS, lysed using the hypotonic buffer and 10% NP-40 and cytoplasmic fraction was collected following the centrifugation step. The remaining nuclear pellet was further treated with the cell extraction buffer and used for analysis. For the whole cell immunoblotting, Cells were lysed with RIPA buffer (50 mM Tris, pH 7.6, 150 mM NaCl, 1% Triton X-100, 0.1% SDS) containing protease and phosphatase inhibitors (#4693159001, Roche, WI, #P0044, Sigma-Aldrich, St. Louis, MO), and processed using lab standard protocol [43]. RAC1-GTP pulldown and analysis were performed using a kit from Cytoskeleton, Inc. (BK035; Denver, CO) according to the manufacturer’s protocol. The antibodies used are listed in Supplementary Table S1D.

### Luciferase assay

A 2562 base pair fragment upstream of *NUMB* gene with enriched binding of RNA Polymerase II and H3K4me3 was identified using Cistrome data and UCSC genome browser (http://cistrome.org/db/#/) in MDA-MB-231 and MCF7. The promoter of *NOTCH1* was identified for MDA-MB-231 cells [44]. The *NUMB* and *NOTCH1* promoter regions were amplified from MDA-MB-231 genomic DNA (Supplementary Table S1E) and sub-cloned into a pGL3-Basic vector (Promega, Madison, WI). MDA-MB-231 and MCF7 (4 × 10^5^ cells) cells with stable RAB4A knockdown or RAB4A overexpression were transiently transfected with either pGL3 control vector or pGL3 expressing *NUMB* and *NOTCH* promoter regions using the Lipofectamine 2000 protocol (ThermoFisher Scientific, USA). A vector expressing Renilla was co-transfected as a control for the normalization of luciferase activity. Luciferase was measured after 24 h of transfection using the Dual-Luciferase Reporter Assay System (#E1960, Promega Pte Ltd) as per the manufacturer’s protocol.

### Tumor sphere formation assay

Sphere formation was used to study the cancer stemness and was performed as described previously [45]. Briefly, cells were seeded at 400 cells/well in DMEM-F12 containing 0.5% methylcellulose (Sigma-Aldrich, MO, USA), B-27, and N2 (Gibco, MD, USA) in low-adherent culture plates (#3474, Corning) and were cultured for two weeks. For serial plating, spheres were treated with StemPro® Accutase® Cell Dissociation Reagent (Gibco) resuspended, and seeded as mentioned above. Sphere count was analyzed using Open CFU software (Geissmann).

### Animals and xenografts

All animals were treated in accordance with the IACUC Guidelines (protocol no. 2021/SHS/1627). All mice were housed in a temperature and light-controlled environment (12 h light, 12 h dark) and were provided food and water *ad libitum*. For all the cell lines tested, 5 × 10^6^ cells, harvested in DMEM containing 10% FBS and 50% Matrigel (BD sciences), were injected subcutaneously into the flank of 8-week-old NOD-SCID-Gamma female mice. Although randomization was not employed in this study, efforts were made to minimize bias by including at least 5 mice per group. When the tumors reached 1 cm^3^ (*L* × *W*^2^/2), the mice were euthanized by CO_2_ inhalation followed by cervical dislocation, and the tumors were removed and processed.

### Statistical analysis

Statistical analysis was carried out using GraphPad Prism software (GraphPad, CA, USA). Data are presented as the mean ± standard error of the mean (SEM) and represent at least three independent biological replicates. Statistical significance was determined by either Student’s unpaired *t*-test, one-way ANOVA, or two-way ANOVA. ANOVAs were followed with Dunnett’s multiple comparisons or Tukey’s post hoc test. *p* < 0.05 is considered significant.

## Results

### Gene expression analysis reveals that RAB4A regulates transcription program(s) controlling cancer stemness through the activation of RAC1

**Fig. 1:**
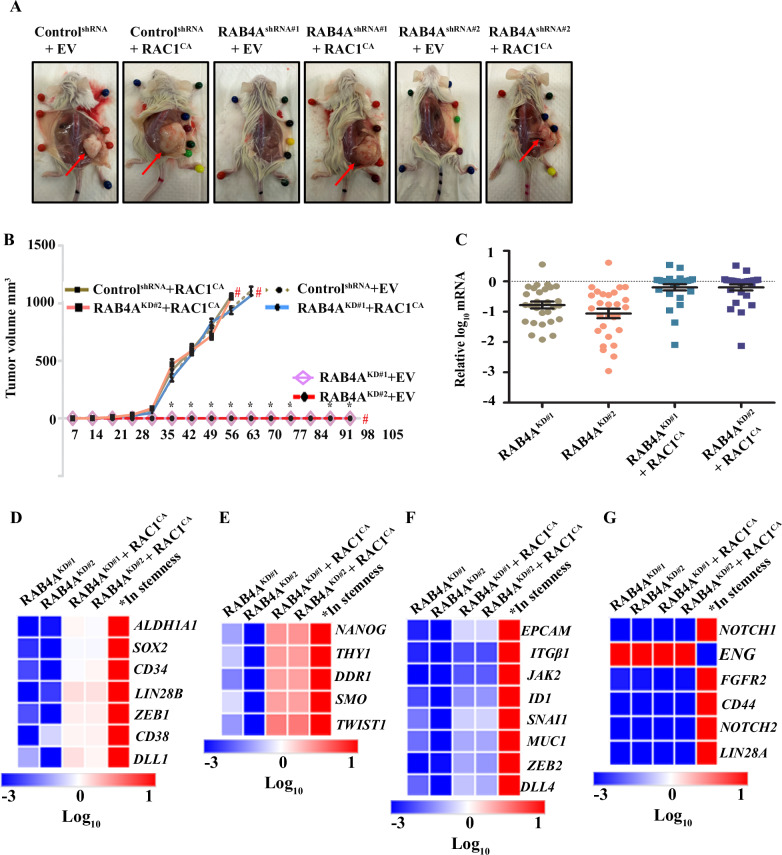
RAB4A is a master regulator of cancer cell stemness. **A**, **B** A subcutaneous xenograft mouse model demonstrates the roles of RAB4A and RAC1 in the regulation of tumor formation. **A** Representative photos of tumors for each group at the endpoint of the experiment; the tumors are derived from MDA-MB-231 cells that express either control shRNA or that target RAB4A with and without concurrent exogenous RAC1-CA expression. **B** Tumor formation and growth for each group as described in (**A**). Prism software was used for the analysis. The group identities and numbers of mice in each group are written in the inset of the figure. **C**–**G** qPCR analysis was performed using prime PCR multiplex assay that contains 87 genes that have been validated for their roles in cancer stemness. Relative mRNA expression was calculated compared to the control cells. Genes that were altered consistently by the two shRNA sequences targeting RAB4A are represented. The heat map was generated using the Morpheus software. The fifth column denotes the direction of the regulation in a typical cancer stemness phenotype based on published research [54–68]. **C** Group scatter plot for the 28 genes that are consistently altered by RAB4A-shRNA#1 and #2. **D**–**G** Heat maps of the gene set that are changed by RAB4A and rescued by RAC1 to different levels: **D** to the base levels, **E** above the base level, **F** below the base level, and **G** not rescued by RAC1.

In our recent studies, we found that RAB4A is essential for the continuous proliferation and maintenance of EMT features in cancer cells of multiple tissue origins [29]. We also discovered that this role of RAB4A acts through controlling RAC1 activation. RAB4A suppression leads to the loss of cancer cell sphere formation in vitro and loss-of-tumor formation in both subcutaneous and orthotopic xenograft mouse models (Fig. 1A, B and Supplementary Fig. S1) [29]; this loss of tumorigenesis can be rescued by exogenous expression of constitutively active RAC1 (RAC1^CA^) (Fig. 1A, B and Supplementary Fig. S1) [29]. Table 1 summarizes the results of the two mouse-model studies.

**Table 1:** The role of RAB4A and RAC1 in tumor formation in orthotopic and subcutaneous mouse models.

	Mammary fat pad model		Subcutaneous model	
Cell lines (derived from MDA-MB-231)	No. of mice	No. of mice formed tumor	No. of mice	No. of mice formed tumor
Control^kd^	15	15 out of 15	5	5 out of 5
Control^kd^ + RAC1^CA^			5	5 out of 5
RAB4A^kd#1^	5	0 out of 5	5	0 out of 5
RAB4A^kd#2^	5	0 out of 5	5	0 out of 5
RAB4A^kd#1^ + RAC1^WT^	5	0 out of 5		
RAB4A^kd#1^ + RAC1^CA^	5	5 out of 5	5	5 out of 5
RAB4A^kd#2^ + RAC1^WT^	5	0 out of 5		
RAB4A^kd#2^ + RAC1^CA^	5	5 out of 5	5	5 out of 5

The effect of RAB4A KD on tumor formation is likely from RAB4A function on stem cell signaling as we have recently found that RAB4A affects EMT. To provide more evidence, we have performed some relevant experiments to rule out the potential effects of RAB4A on proliferation and cell death, which can also contribute to tumor formation. To this end, we observed no effect of RAB4A on cell proliferation nor apoptosis assayed on adherent cultures (Supplementary Fig. S2A, B). Serial-replating sphere formation assay is a commonly used assay to distinguish the short-term effect on proliferation or viability from that on the long-term self-renewal ability of the cells under study. The initial plating, i.e., first-generation sphere formation can be an indicator of the short-term or acute effect on the cells; however, the subsequent replating sphere formation assesses the long-term self-renewal or stemness of the cancer cells. From the serial-replating sphere formation assays on the effect of RAB4A KD, the sphere numbers between control cells and the RAB4A KD cells are distinctly different at third replating (gen-3) but not significantly different at the initial plating (gen-1) (Supplementary Fig. S2C), consistent with the results from viability and proliferation study to support the conclusion that the impact of RAB4A KD is mainly on long term self-renewal ability, i.e., stemness.

To delineate the molecular mechanism(s) accountable for the strong RAB4A–RAC1 signaling in the regulation of stemness and tumorigenesis, we performed a differential gene expression analysis on a group of experimentally validated CSC-related genes (SAB target list H96, Bio-Rad). Specifically, the expression levels of these stem genes were compared among MDA-MB-231 control cells, those stably expressing RAB4A shRNA, and those concurrently expressing RAB4A shRNA and CA-RAC1. Two RAB4A targeting shRNAs were used to assess the effects of RAB4A knockdown, and for each shRNA, CA-RAC1 was introduced to assess the rescue effects. The stable RAB4A knockdown and RAC1 activation status in these cell lines were validated before the gene-expression analysis (Supplementary Fig. S3). Among the 87 genes tested, the expression of 27 showed consistent changes between the RAB4A-shRNA1 and RAB4A-shRNA2 expressing cells and between the two sets of CA-RAC1 rescue cells (Supplementary Table S2 and Supplementary Fig. S4). Strikingly, almost all the genes that were consistently changed were down-regulated upon RAB4A knockdown, demonstrating the central role of RAB4A in maintaining stemness (Fig. 1C). For a large proportion of the down-regulated genes, the expression was reversed by CA-RAC1 expression (Fig. 1D). Further, given that the gene expression changes are under the same RAB4A knockdown and CA-RAC1 expression, the level of CA-RAC1 rescue for each gene provides clues on the extent of RAC1 involvement downstream of RAB4A in regulating stemness/EMT. For the first group of genes, CA-RAC1 expression reverses the expression to near the baseline level, suggesting a linear RAB4A–RAC1 axis as the sole regulation (Fig. 1D). For the second group of genes, CA-RAC1 expression over-compensated the RAB4A knockdown effect, demonstrating a broader and bigger role of RAC1 than that of RAB4A in regulating the expression of the gene (Fig. 1E). For the third group of genes, the CA-RAC1 expression only partially reversed the effect of RAB4A knockdown, suggesting that downstream of RAB4A there may be other regulator(s) besides RAC1 (Fig. 1F). For yet another group, CA-RAC1 expression makes no difference to the RAB4A regulated expression, which implies two possibilities—either these genes are upstream of RAC1 in RAB4A signaling or RAB4A signals through pathway(s) independent of the said gene(s) (Fig. 1G).

In summary, the expression analysis of the select panel of CSC-related genes showed that RAB4A is a master regulator of CSCs and EMT, and that RAC1 is the major downstream mediator for much of this role of RAB4A (Fig. 1C–F). Even for the group of genes that are not rescued by CA-RAC1 expression, they could be upstream of RAC1 in the RAB4A signaling especially considering that they are mostly cell surface proteins.

### RAB4A regulates the expression of critical stem cell factors NUMB, NOTCH, and SOX2

**Fig. 2:**
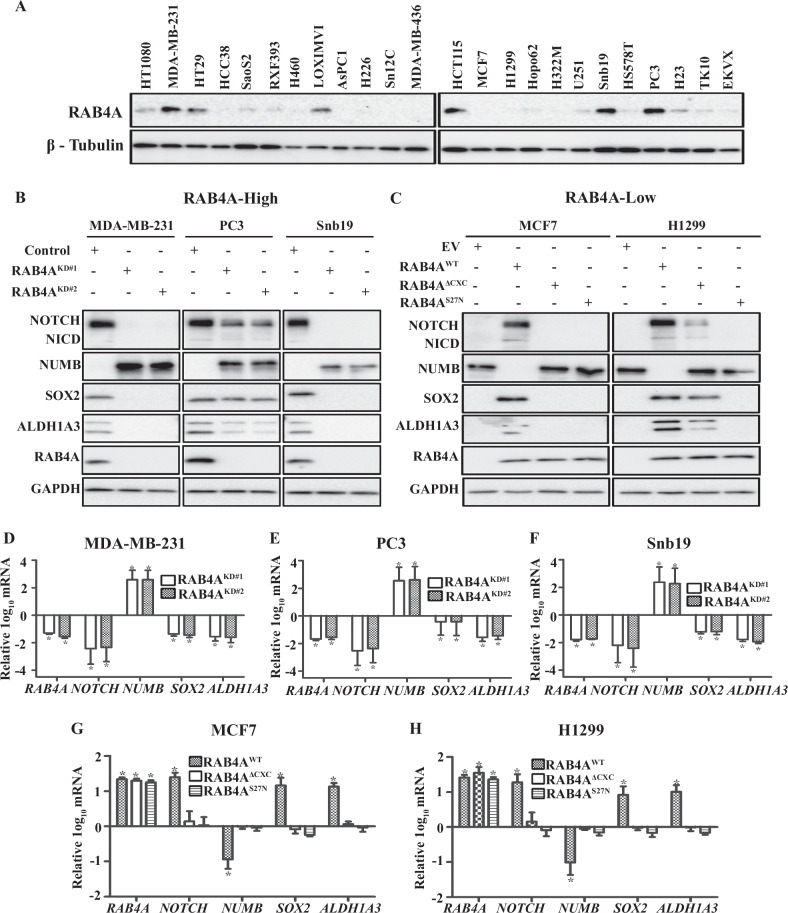
RAB4A promotes the transcription of NOTCH1 and SOX2 while suppressing NUMB transcription. **A** Immunoblot quantification of RAB4A protein levels in a group of cancer cell lines of diverse tissue origins. **B**, **C** Immunoblot analysis of NUMB, NOTCH1/NICD, SOX2, and ALDH1A3 protein levels in response to either RAB4A knockdown or over-expression in multiple cancer cell lines. Cell lysates were prepared for western blot from (**A**) the RAB4A-high MDA-MB-231 breast, PC3 prostate, and SNB19 glioblastoma cells expressing either control shRNA or two different RAB4A targeting shRNAs. Cell lysates were also prepared from (**B**) the RAB4A-low MCF7 breast and H1299 non-small-cell lung cancer cells expressing either wildtype-RAB4A or one of the two mutant RAB4As—RAB4A-ΔCXC with missing C-terminal post-translational modification site and the dominant negative RAB4A(S27N). **D**–**F** qPCR analysis of NOTCH1, NUMB, SOX2, and ALD1A3 transcript levels in RAB4A-high MDA-MB-231 cells (**C**), PC3 cells (**D**), and SNB19 cells (**E**), each expressing either control shRNA or one of the two RAB4A targeting shRNAs. For each gene, the transcript level in the control shRNA-expressing cell is used as the baseline control (the 0 line). **G**, **H** qPCR analysis of NOTCH1, NUMB, SOX2, and ALD1A3 transcript levels in MCF7 cells (**F**) and H1299 cells (**G**) expressing wildtype-RAB4A or either of the two RAB4A mutants—RAB4AΔCXC and RAB4A(S27N), as well as the empty vector control. The expression levels in empty vector control cells are used as the baseline (the 0 line) for each gene. Data are presented as mean ± SEM (*n* ≥ 3); ^*^Indicates when *p* < 0.05 compared to control.

The differential gene expression of CSC regulators in response to RAB4A knockdown and CA-RAC1 expression (Fig. 1) is consistent with the tumorigenesis phenotypes associated with RAB4A and RAC1 functions that we have shown in both previous [29] and current in vitro and in vivo studies (Fig. 1 and Table 1). As the pathway signatures identified RAB4A as a major upstream stem cell regulator, we next explored the changes in the levels of major cancer-driving proteins in MDA-MB-231 control and RAB4A knockdown cells. Among these cancer-signaling proteins, we observed a significant reduction of NOTCH1 and SOX2 levels and, in contrast, a dramatic elevation of NUMB protein levels upon RAB4A knockdown (Supplementary Fig. S5); the NOTCH1 and SOX2 changes are consistent with the gene expression results as presented in Fig. 1. To facilitate the studies on the impact of RAB4A, we assayed a group of cancer cell lines for the expression of RAB4A and categorized them into RAB4A-high and RAB4A-low groups (Fig. 2A). We then validated the levels of NUMB, NOTCH, and SOX2 in a number of RAB4A-high cancer cells (MDA-MB-231, PC3, and SNB19) in response to RAB4A knockdown (Fig. 2B), and in RAB4A-low cancer cells (MCF7 and H1299) in response to RAB4A over-expression (Fig. 2C). The findings confirmed that RAB4A positively controls levels of NOTCH1 and SOX2, and negatively of NUMB, in these cancer cells. We then looked at the transcript levels of these genes and found that, consistent with the protein levels, the transcription of Notch1, Sox2, and the stem cell marker Aldh1a3 genes were suppressed, while that of Numb was elevated, in response to RAB4A knockdown in RAB4A-high cancer cells (Fig. 2D–F). In the RAB4A-low cells, over-expression of wild-type RAB4A, but not the inactive mutant protein RAB4A(S27N) nor the C-terminal deletion form RAB4A(ΔCXC), increased the expression of Notch1, Sox2, and Aldh1a3 while reducing Numb expression (Fig. 2G, H). These observations on both protein and transcript levels suggest that RAB4A controls these cancer stemness regulators mainly through gene expression.

### Constitutively active RAC1 expression reverses the RAB4A knockdown effect on SOX2 but not NUMB or NOTCH1 expression

From our recent report and the above stemness gene expression analysis, we know that RAC1 is a major mediator for RAB4A regulation of EMT and cancer stemness. With the validation in multiple cell lines that RAB4A controls the transcription of Numb, Notch1, and Sox2 genes, we proceeded to study the role of RAC1 on these genes. For this assessment, we performed the protein quantification on nuclear and cytosolic fractions since NUMB, NOTCH1, and SOX2 all have major functions in regulating gene transcription and their cellular localization impacts these functions.

**Fig. 3:**
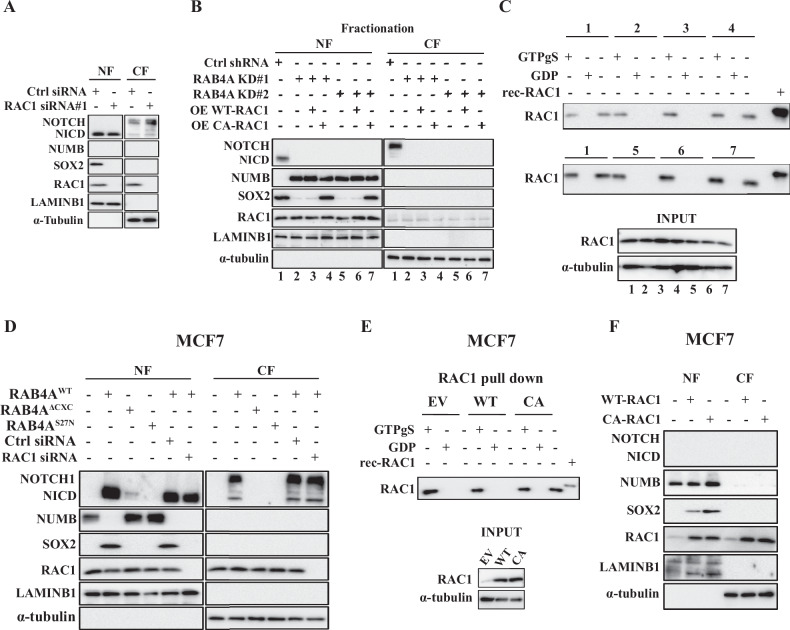
RAC1 has no effect on the expression of NUMB or NOTCH1 but is essential for SOX2 expression. **A** Immunoblot analysis of NUMB, NOTCH1, and SOX2 protein levels in response to RAC1 knockdown in MDA-MB-231 cells. Cell lysates and nuclear/cytosol fractionation were prepared for western blot from MDA-MB-231 cells expressing control siRNA, or RAC1 siRNA. **B** Immunoblot analysis of NUMB, NOTCH1, and SOX2 protein levels in response to either RAB4A knockdown alone or simultaneous RAB4A knockdown and RAC1 over-expression in MDA-MB-231 cells. Cell lysates and nuclear/cytosol fractionation were prepared for western blot from control cells (1), or RAB4A shRNA#1 (2), or RAB4A shRNA#1 and WT-RAC1 cDNA (3), or RAB4A shRNA#1 and CA-RAC1 cDNA (4), or RAB4A shRNA#2 (5), or RAB4A shRNA#2 and WT-RAC1 cDNA (6), or RAB4A shRNA#2 and CA-RAC1 cDNA (7). The label below each lane (1, 2, 3, 4, 5, 6, and 7) marks the identity of the cell lysate as described above and also be used for the pulldown study in (**C**). **C** Pulldown of GTP-bound RAC1 to study the activation status of RAC1 in samples 1, 2, 3, 4, 5, 6, and 7 as described in (**B**). For each condition, the first lane is from the lysate incubated with GTPγS, the second lane is the lysate incubated with GDP, and the third lane is the sample without incubation with nucleotide, therefore assessing the quantity of the GTP bound form of RAC1 in the lysate. At the bottom of the panel is a blot of the lysate without a pull-down to show the quantity of RAC1 in the input. **D** Immunoblot analysis of NUMB, NOTCH1, and SOX2 protein levels in MCF7 cells in response to RAB4A (including WT, ΔCXC, and DN-S27N form of RAB4A) expression and concurrent knockdown of RAC1 in the presence of WT-RAB4A expression. Cell lysates and nuclear/cytosol fractionation were prepared for the western blots. **E** Pulldown of GTP-bound RAC1 to study the activation status of RAC1 in MCF7 cells either with exogenous expression of either WT-RAC1 (2) or CA-RAC1 (3) in comparison to introducing only empty vector (1). For each condition, the first lane is from the lysate incubated with GTPγS, the second lane is the lysate incubated with GDP, and the third lane is the sample without incubation with nucleotide, therefore assessing the quantity of the GTP bound form of RAC1 in the lysate. At the bottom of the panel is a blot of the lysate without a pull-down to show the quantity of RAC1 in the input. **F** Immunoblot analysis of NUMB, NOTCH1, and SOX2 protein levels in MCF7 cells in response to the expression of WT-RAC1 (2) or CA-RAC1 (3) in comparison to the empty vector control (1). Cell lysates and nuclear/cytosol fractionation were prepared for the western blots.

First, we found that, in the RAB4A-high MDA-MB-231 breast cancer cells, NUMB expression is essentially absent and NOTCH1 and SOX2 expression are easily visualized by western blot (Fig. 3A). NICD, the active NOTCH1 fragment, and SOX2 are exclusively localized in the nucleus while the full-length NOTCH1 is mostly in the cytosolic fraction as expected (Fig. 3A). Distinct from the impact of RAB4A knockdown, RAC1 knockdown only abolished the expression of SOX2, but caused no noticeable change in NOTCH1/NICD and NUMB levels (Fig. 3A). We then evaluated the rescue effect of RAC1 over-expression in RAB4A knockdown cells on the levels of these stem cell factors. As presented earlier, RAB4A knockdown completely abolished the NOTCH1/NICD expression and induced NUMB expression that is exclusively localized in the nucleus (Fig. 3B). Notably, neither NOTCH nor NUMB levels were affected by exogenous WT- or CA-RAC1 expression (Fig. 3B). In contrast, the expression of SOX2, lost from RAB4A knockdown was restored by CA-RAC1 but not WT-RAC1 (Fig. 3B). Consistently, pulldown assays using p21 binding domain (PBD, which pulls down only the activated RAC1/GTP-bound RAC1) confirmed that only the expression of CA-RAC1 but not WT-RAC1 increases the level of GTP-bound RAC1 in MDA-MB-231 cells (Fig. 3C).

The impact of RAB4A and RAC1 was also evaluated in the RAB4A-low breast cancer cell line MCF7. In this case, we observed high baseline NUMB and low baseline NOTCH and SOX2 protein levels (Fig. 3D), opposite to those in the RAB4A-high cells. When WT-RAB4A was expressed, the NUMB level was suppressed while the NOTCH1 and SOX2 levels were induced; these effects of WT-RAB4A were obliterated by RAC1 knockdown (Fig. 3D). The effects on NUMB, NOTCH1 and SOX2 by WT-RAB4A were not observed by the expression of either RAB4A(S27N) or RAB4A(ΔCXC) (Fig. 3D), consistent with the notion that the C-terminal lipid modification site and the activation status of RAB4A are necessary for its regulation of stemness.

Following the RAB4A expression study, we also assessed the effects of introducing RAC1 in MCF7 cells. In this case, we compared the control cells (empty expression vector) with those cells expressing either WT-RAC1 or CA-RAC1 proteins. Validating with the PBD pulldown RAC1 activity assay, we found that there is little activation of RAC1 in MCF7 cells at baseline, in contrast to the high activation level in MDA-MB-231 cells, despite the presence of similar levels of total RAC1 protein (Fig. 3E). This observation is consistent with the role of RAB4A in regulating RAC1 activation [42]. The pulldown assay also confirmed that expression of CA-RAC1 dramatically increased the level of activated RAC1 while WT-RAC1 contributes little to the activity of RAC1 (Fig. 3E). CA-RAC1 had no effect on NUMB and NOTCH1 protein levels while caused significant increase in SOX2 level (Fig. 3F), consistent with the hypothesis that RAC1 is upstream of SOX2 in the RAB4A signaling chain. Notably, WT-RAC1 increases SOX2 at a much-attenuated level compared to CA-RAC1 (Fig. 3F), consistent with the conclusion from MDA-MB-231 cells (Fig. 3B) that the activation is required for RAC1 to increase breast cancer cell stemness. In conclusion, these studies in both RAB4A-high and RAB4A-low cancer cells demonstrate that RAC1 is upstream of SOX2 in the RAB4A regulation of cancer stemness, while the order of regulation among RAC1, NUMB, and NOTCH1 are yet to be defined.

### Suppressing NUMB expression reverses the effects of RAB4A knockdown on NOTCH1 and SOX2 expression, RAC1 activity, and the stemness phenotype

**Fig. 4:**
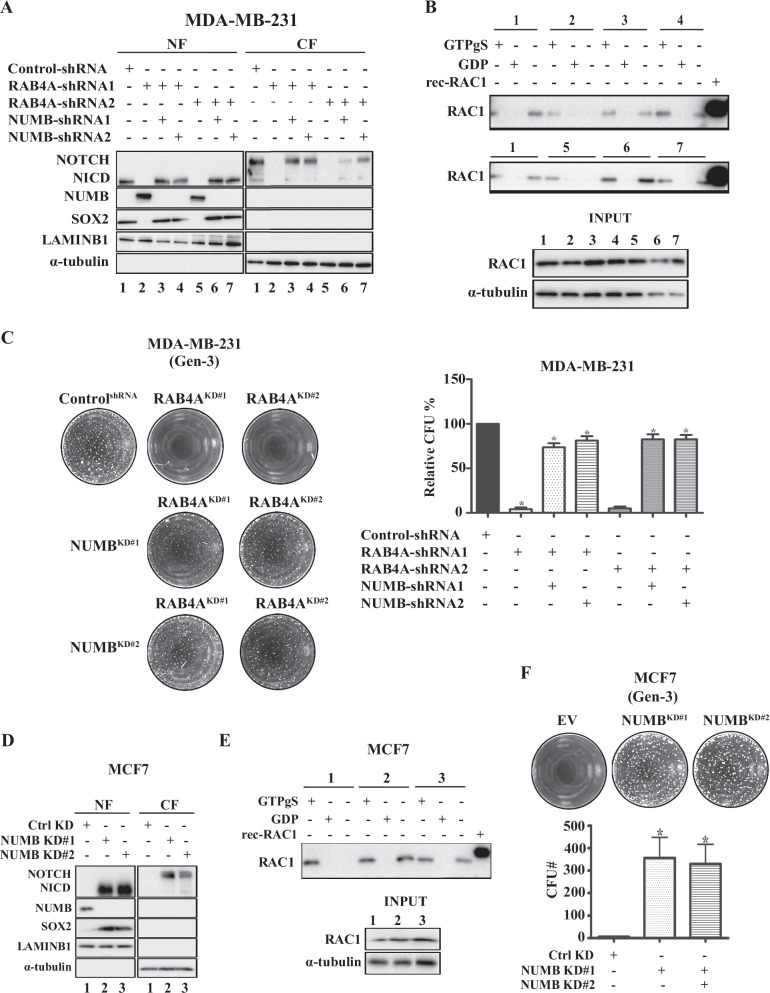
RAB4A regulates cancer stemness through suppression of NUMB, which in turn modulates NOTCH1 and SOX2 expression and RAC1 activation. **A** Immunoblot analysis of NUMB, NOTCH1, and SOX2 protein levels in response to RAB4A knockdown alone and combined RAB4A and NUMB knockdown in MDA-MB-231 cells. Cell lysates and nuclear/cytosol fractionation were prepared for western blot from MDA-MB-231 cells expressing control shRNA (1), or RAB4A shRNA#1 (2), or concurrently RAB4A shRNA#1 and NUMB shRNA#1 (3), or concurrently RAB4A shRNA#1 and NUMB shRNA#2 (4), or RAB4A shRNA#2 (5), or concurrently RAB4A shRNA#2 and NUMB shRNA#1 (6), or lastly RAB4A shRNA#2 and NUMB shRNA#2 (7). The numeric label below each lane indicates the identity of the cell lysate as described above and will be used in panel (**B**) pulldown assay. **B** Pulldown of GTP-bound RAC1 to study the activation status of RAC1 in samples 1, 2, 3, 4, 5, 6, and 7 as described in (**A**). For each condition, the first lane is from the lysate incubated with GTPγS, the second lane is the lysate incubated with GDP, and the third lane is the sample without incubation with nucleotide to assess the quantity of the GTP bound form of RAC1 in the lysate. At the bottom of the panel is a blot of the lysate input. **C** Serial replating sphere formation assay on MDA-MB-231 cells with RAB4A knockdown alone and in combination with NUMB knockdown. The left side of the panel shows the representative microscopic images of the third-generation (Gen-3) plated spheres; the right side of the panel presents the quantification of spheres. **D** Immunoblot analysis of NUMB, NOTCH1, and SOX2 protein levels in response to NUMB knockdown in MCF7 cells. Cell lysates and nuclear/cytosol fractionation were prepared for western blot from cells expressing control shRNA (1), or NUMB shRNA#1 (2), and NUMB shRNA#2 (3). The numeric label below each lane indicates the identity of the cell lysate as described and will be used in (**E**). **E** Pulldown of GTP-bound RAC1 to study the activation status of RAC1 in samples 1, 2, and 3 as described in Panel (**D**). For each condition, the first lane is from the lysate incubated with GTPγS, the second lane is the lysate incubated with GDP, and the third lane is the sample without incubation with nucleotide. At the bottom of the panel is a blot of the lysate input. **F** Serial replating sphere formation assay on MCF7 cells with NUMB knockdown. The top of the panel shows the representative microscopic images of the third-generation (Gen-3) plated spheres; the bottom of the panel presents the quantification of spheres. Data from sphere formation assays (**C**, **F**) are presented as mean ± SEM (*n* ≥ 3); ^*^Indicates when *p* < 0.05 compared to control.

We have presented evidence that links NUMB to RAB4A regulation for cancer cell stemness (Figs. 2 and 3), and that loss-of-RAB4A leads to an increased expression of NUMB protein in RAB4A-high cancer cells and exogenous expression of RAB4A in RAB4A-low cells suppresses NUMB protein (Fig. 3). We also found that RAC1 regulates the expression of SOX2 but not NUMB or NOTCH1. To further delineate this signaling pathway, the role of NUMB regulation in the RAB4A–RAC1 axis of signaling was evaluated. To this end, we assessed the effect of the simultaneous knockdown of RAB4A and NUMB in MDA-MB-231 cells. Here we observed that, by knocking down NUMB, the effects of RAB4A knockdown on the suppression of NOTCH1 and SOX2 expression were reversed, suggesting that NUMB is upstream of these two proteins in the RAB4A regulation of stemness (Fig. 4A). We also found, in this setting, that RAC1 activity was restored with NUMB knockdown (Fig. 4B). Phenotypically, supressing NUMB restored the serial replating sphere formation ability that was lost due to RAB4A knockdown (Fig. 4C), demonstrating the essential role of NUMB in RAB4A regulation of self-renewal.

The relevance of NUMB is also evaluated in the RAB4A-low, NUMB-high MCF7 breast cancer cells. Here, the knockdown of NUMB increased NICD/NOTCH1 and SOX2 protein levels, with SOX2 and NICD mostly in the nuclear fraction, consistent with the findings from MDA-MB-231 cells (Fig. 4D). RAC1 activity assay shows that knockdown NUMB increased GTP-bound RAC1 level, consistent with the notion that NUMB as an upstream regulator of RAC1 (Fig. 4E). Serial replating sphere formation assay showed that suppressing NUMB dramatically increased the self-renewal ability of MCF7 from the baseline (Fig. 4F). In conclusion, we present strong evidence to support that, in the RAB4A signaling chain of stemness regulation, NUMB is downstream of RAB4A but upstream of NOTCH1, RAC1, and SOX2. Phenotypically, in both RAB4A high/NUMB low and RAB4A low/NUMB high cancer cells, NUMB suppresses the long-term sphere formation—a hallmark of cancer stemness.

### NOTCH1 is upstream of RAC1 and SOX2 in the RAB4A–NUMB signaling pathway that promotes cancer stemness

**Fig. 5:**
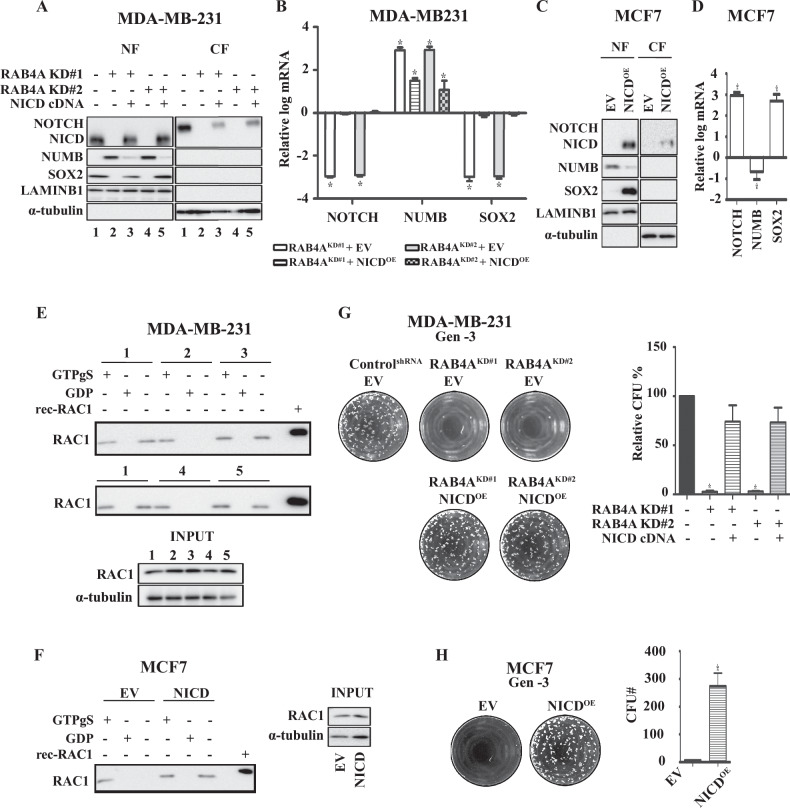
NICD expression increases RAC1 activation and SOX2 level, and restores the sphere-forming ability that was lost from RAB4A knockdown. **A** Immunoblot analysis of NUMB, NOTCH1, and SOX2 protein levels in response to NICD expression in MDA-MB-231 cells. Cell lysates and nuclear/cytosol fractionation were prepared for western blot from MDA-MB-231 cells expressing control shRNA (1), or RAB4A shRNA#1 (2), or concurrently RAB4A shRNA#1 and NICD cDNA (3), or RAB4A shRNA#2 (4), or concurrently RAB4A shRNA#2 and NICD cDNA (5). The numeric label below each lane indicates the identity of the cell lysate as described above. The sample identities are used in the pulldown assay of panel E. **B** qPCR analysis of NOTCH1, NUMB, and SOX2 expression in the cells as described in (**A**). The control shRNA cell expression levels are used as the baseline control for each gene. **C** Immunoblot analysis of NUMB, NOTCH1, and SOX2 protein levels in response to NICD expression in MCF7 cells. Cell lysates and nuclear/cytosol fractionation were prepared for western blot from MCF7 cells with or without exogenous NICD expression. **D** qPCR analysis of NOTCH1, NUMB, and SOX2 expression in the cells as described in (**C**). The baseline is set by the expression of each gene in MCF7 cells without the exogenous expression of NICD. **E** Pulldown of GTP-bound RAC1 to study the activation status of RAC1 in samples 1, 2, 3, 4, and 5 as described in (**A**). For each condition, the first lane is from the lysate incubated with GTPγS, the second lane is the lysate incubated with GDP, and the third lane is the sample without incubation with nucleotide. At the bottom is a blot of the lysate input. **F** Pulldown of GTP-bound RAC1 to study the activation status of RAC1 of the lysate from MCF7 cells with or without exogenous expression of NICD. **G** Serial replating sphere formation assay on MDA-MB-231 cells with RAB4A knockdown alone and in combination with NICD overexpression. The left side of the panel shows the representative microscopic images of the third-generation (Gen-3) plated spheres; the right side of the panel presents the quantification of spheres. **H** Serial replating sphere formation assay on MCF7 cells with and without exogenous NICD expression. The left side images are representative microscopic photos and on the right it shows the analysis. Data from **B**, **D**, **G**, and **H** are from three biological repeats and are presented as mean ± SEM (*n* ≥ 3); ^*^When *p* < 0.05 compared to control.

The data presented thus far demonstrates that NUMB is essential for RAB4A regulation of stemness, and that it does so through NOTCH1, RAC1, and SOX2. Here we further assessed the position of NOTCH1 in the signaling chain of RAB4A regulation. To this end, we performed a rescue experiment by expressing NICD—the transcription activator fragment of NOTCH1—in RAB4A knockdown MDA-MB-231 cells. When exogenous NICD was introduced in the RAB4A knockdown cells, the SOX2 level recovered to the comparable level as control cells (Fig. 5A). From the qPCR analysis, we observed that NICD expression restored the transcript level of SOX2, consistent with the role of NICD in the transcription regulation (Fig. 5B) [46, 47]. We also noted a slight increase in the full-length NOTCH1 protein, supporting the notion that there exists some level of self-regulation of NOTCH1. Interestingly, we observe that NICD expression also affected NUMB at a modest level, suggesting a mutual regulatory mechanism between NUMB and NOTCH1 (Fig. 5A, B), albeit the NUMB control of NOTCH expression appears to be the dominant one (Fig. 4A, D). We also introduced NICD in the NUMB-high, NOTCH1-low MCF7 cells; here the expression of NICD elicited a strong SOX2 expression and a modest suppression of NUMB (Fig. 5C, D), consistent with the observations made in MDA-MB-231 cells. To place the order of NICD and RAC1 in RAB4A signaling, we evaluated RAC1 activity level in relation to NICD level and found that increasing NICD enhanced RAC1 activity (Fig. 5E, F), suggesting that NICD is a strong positive regulator of RAC1 activation.

We then evaluated the phenotypic impact of NICD in RAB4A regulation of cancer cell stemness. As expected, knockdown RAB4A in high sphere-forming MDA-MB-231 cells abolished the third passage sphere formation, which was rescued by the expression of NICD to similar levels as the parental cells (Fig. 5G). For the low sphere-forming MCF7 cells, NICD expression dramatically increased the third-generation sphere formation (Fig. 5H). In summary, these studies conclude that NOTCH1, through its activation product NICD, forms a necessary link in the RAB4A signaling axis upstream of RAC1 and SOX2 in promoting the cancer cell stemness/self-renewal property. NUMB and NICD also appear to form a mutual regulation loop to exert a balance in controlling stemness.

### SOX2 is downstream of NUMB, NOTCH1, and RAC1 in mediating the signal from RAB4A in the regulation of cancer cell stemness/self-renewal

**Fig. 6:**
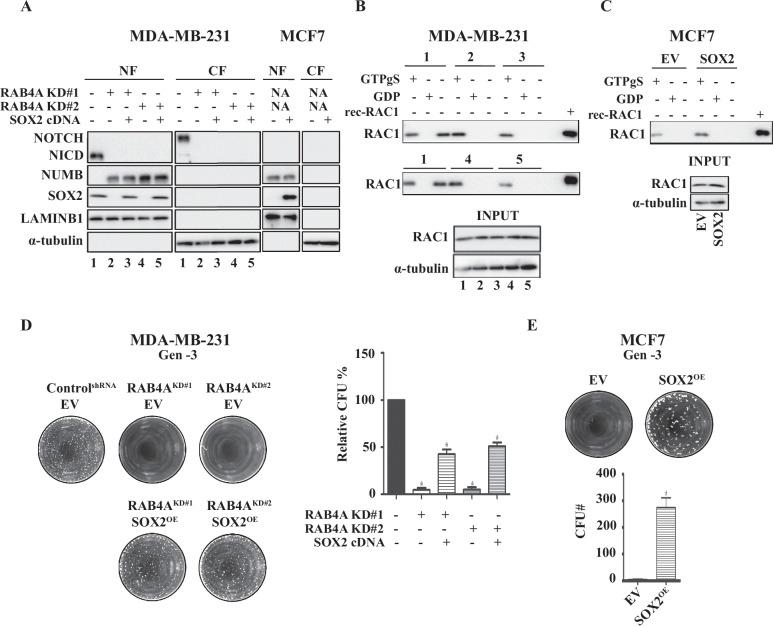
SOX2 expression restores the sphere-forming ability that was lost from RAB4A knockdown. **A** Immunoblot analysis of NUMB, NOTCH1, and SOX2 protein levels in response to SOX2 expression in MDA-MB-231 and MCF7 cells. For MDA-MB-231 cells, the lysates and nuclear/cytosol fractionation were prepared for western blot from cells expressing control shRNA (1), or RAB4A shRNA#1 (2), or concurrently RAB4A shRNA#1 and SOX2 cDNA (3), or RAB4A shRNA#2 (4), or concurrently RAB4A shRNA#2 and SOX2 cDNA (5). The label below each lane indicates the identity of the cell lysate as described above. For MCF7 cells, the lysates and fractionation were prepared from cells with and without exogenous SOX2 expression. **B** Pulldown of GTP-bound RAC1 to study the activation status of RAC1 in samples 1, 2, 3, 4, and 5 as described in (**A**). For each condition, the first lane is from the lysate incubated with GTPγS, the second lane is the lysate incubated with GDP, and the third lane is the sample without incubation with nucleotide, therefore assessing the quantity of the GTP bound form of RAC1 in the lysate. At the bottom of the panel is a blot of the lysate without a pull-down to show the quantity of RAC1 in the input. **C** Pulldown of GTP-bound RAC1 to study the activation status of RAC1 of the lysate from MCF7 cells with or without exogenous expression of SOX2. **D** Serial replating sphere formation assay on MDA-MB-231 cells with RAB4A knockdown alone and in combination with exogenous SOX2 expression. The left side of the panel shows the representative microscopic images of the third-generation (Gen-3) plated spheres; the right side of the panel presents the quantification of spheres. **E** Serial replating sphere formation assay on MCF7 cells with and without exogenous SOX2 expression. The top images are representative microscopic photos and the bottom shows the analysis from three biological repeats of the third generation sphere. Data analysis of (**D**, **E**) are from three biological repeats and presented as mean ± SEM (*n* ≥ 3); ^*^Indicates when *p* < 0.05 compared to control.

So far, we have demonstrated that NUMB, NOTCH1, and RAC1 regulate cancer cell self-renewal downstream of RAB4A, and that this signaling chain controls the transcription of SOX2. However, the question of whether SOX2 is necessary and sufficient in the RAB4A regulation of cancer stemness still needs to be addressed. To this end, we performed a SOX2 rescue study in MDA-MB-231 RAB4A knockdown cells, and in RAB4A-low MCF7 parental cells, to determine whether the self-renewal could be boosted. We found that, introducing physiological levels of SOX2 expression exerted no impact on the levels of NUMB and NOTCH1 (Fig. 6A). The RAC1 activity assay demonstrated that SOX2 expression did not change the activation level of RAC1 in either MDA-MB-231 cells (Fig. 6B) or in MCF7 cells (Fig. 6C). From the sphere formation study, we found that SOX2 expression significantly rescued the sphere forming ability that was lost upon RAB4A knockdown in MDA-MB-231 cells (Fig. 6D). In MCF7 cells, SOX2 expression dramatically elevated sphere formation from the low level seen in parental cells (Fig. 6E). It was noted however that, distinct from the NUMB knockdown and NICD expression rescue in MDA-MB-231 cells that almost completely restored the sphere formation ability to the level before RAB4A knockdown (Figs. 4C and 5G), SOX2 expression partially rescued the sphere formation despite the restoration of SOX2 level to that similar to the parental cells (Fig. 6D). These observations suggest that downstream of RAB4A–RAC1 axis of signaling there may be additional mediator(s) besides/parallel to SOX2 to regulate self-renewal. Future studies should shed light on these additional signaling events.

### RAB4A–NUMB–NOTCH1–RAC1–SOX2 signaling is essential for tumor formation in vivo

**Fig. 7:**
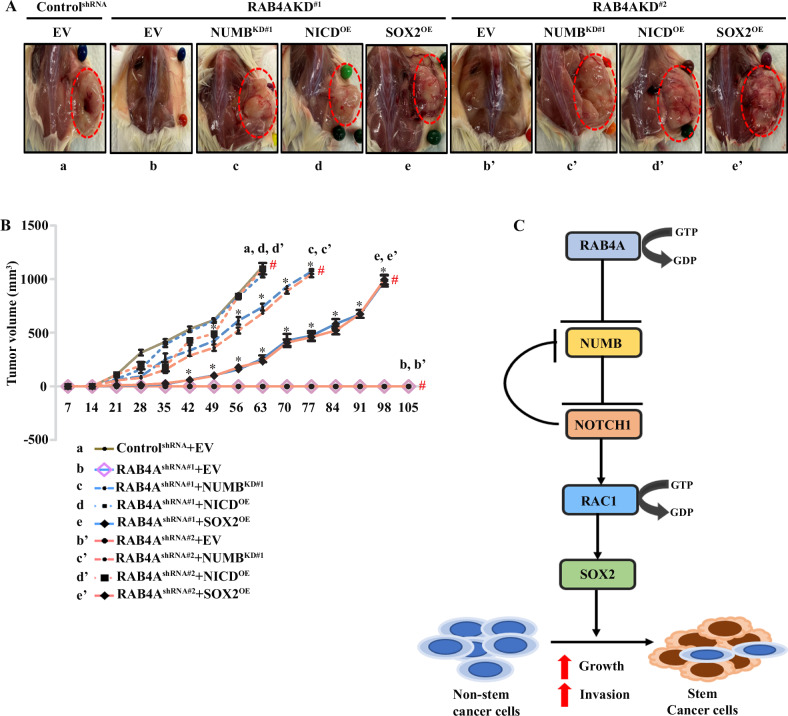
RAB4A–NUMB–NOTCH1–RAC1–SOX2 axis of signaling is essential for tumor formation. **A** Subcutaneous xenograft tumor formation study with NOD SCID mice. This panel shows the photographs of representative mice and the tumor (in red dash circle) at the time of sacrifice. Nine MDA-MB231-derived cell lines were individually injected in NOD SCID mice as described in detail in “Materials and methods”. Five mice were implanted for each cell line (*N* = 5). These cell-line-derived tumors were labeled below the representative tumor images and in the graph for ease to follow. The group identifiers are used below the images. They are, respectively, cells expressing: control shRNA and empty cDNA (EV) vectors (a), RAB4A shRNA#1 and EV (b), RAB4A shRNA#2 and EV (b’), RAB4A shRNA#1 and NUMB shRNA (c), RAB4A shRNA#2 and NUMB shRNA (c’), RAB4A shRNA#1 and NICD cDNA (d), RAB4A shRNA#2 and NICD cDNA (d’), RAB4A shRNA#1 and SOX2 cDNA (e), and RAB4A shRNA#2 and SOX2 cDNA (e’). The red arrow points to the tumor. **B** The tumor growth curves and statistical analysis for the xenograft tumors as described in Panel A. The same group identifiers as in (**A**) are used for each group. “#” marks the time when the mice were sacrificed according to IACUC protocol when tumor size reached 1 cm^3^, or in the case of “b” and “b’” groups it indicated the end of the experiment. The statistical significance was calculated between the control shRNA and EV—group “a” and each other group. ^*^Represents when *p* < 0.05. **C** Graphic description of the signaling chain of RAB4A–NUMB–NOTCH–RAC1–SOX2 that determines cancer stemness.

The in vitro study of the signaling chain whereby RAB4A impacts cancer cell stemness has demonstrated that NUMB, NOTCH1, RAC1, and SOX2 are essential in mediating the regulatory effects of RAB4A on CSCs in multiple cancer cell lines of diverse tissue origins. Among these effects, we have already validated in the in vivo setting that constitutively active RAC1 is able to restore the lost tumor formation ability resulting from RAB4A knockdown [29]. Here, we sought to validate, in a xenograft mouse model, the essential role of NUMB, NOTCH1, and SOX2 in RAB4A-driven tumor formation. From parental MDA-MB-231 cells, we generated stable cell lines that contain the vector of (i) control, (ii) RAB4A shRNA, (iii) RAB4A shRNA and NUMB shRNA, (iv) RAB4A shRNA and NICD cDNA, and (v) RAB4A shRNA and SOX2 cDNA. The identities of these cells were confirmed by gene expression changes (Supplementary Fig. S6). These cells were deposited subcutaneously into the flanks of NOD SCID mice to observe tumor growth. All the mice were sacrificed when the tumors reached around 1 cm^3^ according to the approved IACUC protocol; the representative images for each group are presented in Fig. 7A. Consistent with previous in vivo observation, effective RAB4A knockdown with either of the two shRNAs abolished the tumor-forming ability of MDA-MB-231 cells (Fig. 7A, B). Concurrent knockdown of NUMB largely restored the tumor formation, albeit with a 7- to 10-day delay compared to control cells (Fig. 7B). Expression of NICD, the nuclear fragment of NOTCH1 that is shown to be downstream of NUMB but also feeds back to NUMB, completely reversed the effect of RAB4A knockdown on the tumor forming ability (Fig. 7B). Similar to the observation made in the in vitro sphere formation assay, the expression of SOX2 partially but significantly restored the tumor forming ability, with about a 50-day delay in tumor growth (Fig. 7B). These in vivo rescue experiments confirm the signaling axis of RAB4A–NUMB–NOTCH–RAC1–SOX2 in the control of stemness/self-renewal that is the foundation for tumor formation (Fig. 7C). It is important to point out the discrepancy between the extent of rescue by SOX2 and NICD expression and NUMB knockdown, as well as CA-RAC1 expression [29], which suggests that there may be other regulator(s) acting parallel of SOX2 downstream of NUMB–NOTCH1–RAC1. Further investigation will likely complete this regulatory map.

## Discussion

### RAB4A is a master regulator of tumorigenesis and cancer progression

RAB4A, a member of the RAB family of small GTPases, is well-recognized for its essential function in regulating intracellular vesicle trafficking, particularly the short-loop endosome recycling, through which it plays important roles in cellular function [48]. Relatively, RAB4A is less known for its role in cancer cell signaling; however, several functions have recently emerged in cancer invasiveness and growth. As a regulator of vesicular trafficking, RAB4A may contribute to tumor growth by modulating the transport of various signaling molecules such as growth factors and receptors to the cell surface, thereby affecting cancer cell proliferation and tumor progression [49, 50]. Our recent studies suggest that RAB4A influences the rearrangement of the cytoskeleton, affects the trafficking of integrins and cell adhesion properties, and regulates the EMT process which is considered the common foundation for cancer stemness and metastatic potential [29, 42]. Interestingly, a genetic mouse model demonstrated that the knockout of constitutively active RAB4A resulted in a depletion of immune cells and impaired receptor recycling [51]. Hence, we speculate that RAB4A likely has immune regulatory function and may impact on tumor microenvironment, a major focus in cancer therapy development for many solid tumors.

Despite these recent findings, there remains a major gap in knowledge of the molecular mechanisms underlying the roles of RAB4A in cancer. The work presented in this manuscript describes a step-by-step identification of a novel signaling axis RAB4A–NUMB–NOTCH1–RAC1–SOX2 to elucidate the transmission of signaling downstream from RAB4A to control stemness and tumor formation. Given its impact on cancer stemness and EMT, we postulate that suppressing RAB4A expression or inhibiting its function would likely halt tumor growth and metastasis, and hence is worthy of future evaluation as a therapeutic target. In this current study, we provide direct evidence, using both RAB4A-high and RAB4A-low cancer cell lines of diverse tissue origin, to demonstrate that suppressing RAB4A reduces the oncogenic potential in RAB4A-high cells, and that increasing the level of RAB4A promotes oncogenic potential in RAB4A-low cells. Furthermore, we conclude that the signaling axis of RAB4A–NUMB–NOTCH1–RAC1–SOX2—a previously unknown pathway—is the molecular mechanism important for tumorigenesis.

### Transcription regulation is the predominant mechanism of the RAB4A-to-SOX2 chain of signal transmission

The RAB4A small GTPase is best known for its role in endocytic vesicle recycling, especially that of the so-called short-loop-recycling, transporting endocytosed cargos quickly back to the plasma membrane. In our recent study, we observed that integrin β3 recycling, and in turn its intracellular signaling, is subjected to RAB4A regulation [42]. Hence, it is interesting that we found in this study that RAB4A plays a determinant role in NUMB transcription through which it controls NUMB protein level (Fig. 2). To evaluate whether the RAB4A regulation of NUMB expression is at the transcription or at the post-transcriptional processing level, we performed the classical Actinomycin D treatment study. Consistent with a role on transcription, we found that in the presence of Actinomycin D, the baseline NUMB transcript level is reduced but the half-life of the transcript remains the same with and without RAB4A knockdown (Supplementary Fig. S7A). Notably, this effect is likely not directly downstream of RAB4A but through intermediates, therefore warranting further studies to define.

We also found that NUMB regulates the expression level of NOTCH1 at the transcript level (Fig. 2D–H). Interestingly, prior to our findings, numerous reports described NUMB regulates NOTCH1 trafficking and degradation in the endosome; however, the role of NUMB in NOTCH1 transcription has not been described [52, 53]. Hence, we further investigated the role of NUMB in regulating NOTCH expression by both Actinomycin D treatment and luciferase reporter assays for the impact of both RAB4A and NUMB on NOTCH1 promoter transcription [44]. We found that RAB4A knockdown led to a reduction of basal NOTCH transcript level that was reversed by concurrent knockdown of NUMB. However, neither RAB4A knockdown alone nor combined RAB4A and NUMB knockdown affected the half-life of the NOTCH1 transcript (Supplementary Fig. S7B). These data suggest transcription regulation of NOTCH1 by RAB4A through NUMB. Luciferase reporter assay using NOTCH1 promoter with control KD, RAB4A KD alone, and concurrent RAB4A and NUMB KD provides further support for this notion (Supplementary Fig. S7C, D). To look further into the contribution of endosome-related mechanisms in the regulation of NUMB and NOTCH, we evaluated the impact of knocking down RAB5A on the levels of NUMB, NOTCH/NICD, and SOX2. Since RAB5A is the “yin” to the RAB4A “yang” in endocytosis, we would expect a change in the level of these proteins if endocytosis plays a significant role in their levels. Here, though, we did not observe any effect of RAB5A on the levels of NUMB, NOTCH, and SOX2 (Supplementary Fig. S8). These studies support the conclusion that the RAB4A–NUMB regulation of NOTCH1 is mainly through transcriptional control. We anticipate that future investigation will provide further details on the nature of NUMB control of transcription of NOTCH1.

We acknowledge that the RAB4A–NUMB–NOTCH1–RAC1–SOX2 signaling axis as detailed in this work is not linear at every step. There clearly exists a mutual regulation between NUMB and NOTCH1 in that, although the predominant regulation is transmitted from NUMB to NOTCH1, the expression of NOTCH1 at the physiological level also exerts a negative feedback on NUMB. Notably, the mutual regulation is both at the transcriptional level, which will be an interesting subject in future studies.

### SOX2 is likely not the only effector downstream of RAB4A–NUMB–NOTCH1–RAC1 signaling in the regulation of stemness

Finally, our data point to the existence of other effector(s) downstream of RAC1 besides SOX2 in the control of stemness. This assessment is based on the rescue studies that position the up- and downstream effectors in this pathway, in which we observed that NUMB knockdown and NICD expression nearly completely reversed the loss-of-function phenotypes of RAB4A knockdown both in vitro and in vivo and restored RAC1 activation to the original level. In this regard, in our recent study, we also found that expressing activated RAC1 completely restored the sphere formation and tumor formation ability lost due to loss-of-RAB4A [29]. In contrast, restoring SOX2 expression back to the baseline level of that before RAB4A knockdown only partially rescued the serial-replating sphere formation and tumor formation, which suggests that downstream of RAC1 the regulation of stemness is likely transmitted through multiple effectors, with SOX2 being a predominant one. It will be interesting to identify in future studies other RAC1 downstream effectors that transmit signals for cancer stemness regulation.

Overall, this study identified a novel regulatory axis, RAB4A–NUMB–NOTCH1–RAC1 in regulating cancer stemness and tumor initiation and progression and these targets may be further investigated for drug development and cancer therapeutics.

## Supplementary information

Supplementary Information

Full Blots

## Data Availability

The data supporting the findings of this study are available from the corresponding author upon reasonable request.
