# Supplementary material for: RAB4A is a master regulator of cancer cell stemness upstream of NUMB–NOTCH signaling
Source: Cell Death Dis. 2024 Oct 27;15(10):778. doi: 10.1038/s41419-024-07172-w (PMC11514220; doi:10.1038/s41419-024-07172-w)
Supplement: Supplementary file 1 — Supplementary Information [file 41419_2024_7172_MOESM1_ESM.pdf]

Supplementary Information

Supplementary tables

Supplementary Table S1

Supplementary Table S1A - ShRNA expression vectors

S.No	Description	Vector	Clone ID
1	*RAB4A* human CDS	pLKO_TRC005	TRCN0000232000
2	*RAB4A* human 3' UTR	pLKO_TRC005	TRCN0000232001
3	*NUMB* human CDS	pLKO.1-CMV-neo	TRCN0000063938
4	*NUMB* human 3' UTR	pLKO.1-CMV-neo	TRCN000007223

Supplementary Table S1B - Cloning primers

Gene	Forward primer (5'-3')	Reverse primer (5'-3')	Vector
NICD	ATGCGCCGGCGGCAGCATGGC	TTACTTGAAGGCCTCCGGAATGC	pMSCV-Blasticidin
SOX2	ATGTACAACATGATGGAGACGGAGCT	TCACATGTGTGAGAGGGGCAGT	pCDNA-G418

Supplementary Table S1C - qPCR primers

Gene	Forward primer (5'-3')	Reverse primer (5'-3')
NOTCH	TCCAGGAAACAACTGCAAGAA	TACAGTACTGACCTGTCCACTC
NICD	CTTCCAGATCCTGATCCGG	GGTGTCTCCTCCCTGTTGT
NUMB	GTTAAGTACCTTGGCCATGTAGA	GCCTTTGAAGAACTTCCTTTCAG
SOX2	GCTACAGCATGATGCAGGACCA	TCTGCGAGCTGGTCATGGAGTT
ALDH1A3	GAGCGAATAGCACCGACTATG	CAGTTGATCCAGACCGTTCC

Supplementary Table S1D - Antibodies

Antibody	Cat No	Manufacturer
NOTCH	3608	Cell Signalling
NUMB	ab4147	Abcam
SOX2	MAB2018	Thermo Scientific
ALDH1A3	GTX110784	Genetex
GAPDH	2118	Cell Signalling
LaminB1	AB16048	Abcam
PARP	9542	Cell Signalling
Caspase-3	9662	Cell Signalling
α-tubulin	T9026	Sigma Aldrich

Supplementary Table S1E - Promoter primers

Gene	Forward primer (5'-3')	Reverse primer (5'-3')
NOTCH1	GCGCAGGGACTATGGCAGG	GCCTCCCCACCGGCTG
NUMB	TATAAATTACTAATAAATCACAATCCACA	ATTGAGGCTCCTGGAAAGAA

Supplementary Table S2. Raw Cq values for the cancer stem cell panel PCR

S.No	Gene	Control	$RAB4A^{KD\#1}$	$RAB4A^{KD\#2}$	$RAB4A^{KD\#1}$ $+RAC1^{CA}$	$RAB4A^{KD\#2}$ $+RAC1^{CA}$
1	*ABCB5*	36.65	35.93	37.73	35.70	37.47
2	*B2M*	17.80	18.17	17.96	17.71	17.84
3	*DDR1*	28.44	33.00	35.33	28.34	28.40
4	*ERBB2*	25.17	25.29	24.95	25.12	25.21
5	*GSK3B*	23.66	23.41	23.18	23.61	23.67
6	*ITGB1*	18.36	18.85	18.82	18.33	18.41
7	*LIN28A*	32.65	30.98	30.48	30.81	30.97
8	*NANOG*	25.59	30.31	33.67	25.59	25.58
9	*PROM1*	35.75	35.26	36.36	35.38	35.88
10	*SOX2*	24.47	28.77	29.23	25.31	25.40
11	*WNT1*	36.63	36.01	36.04	33.47	36.76
12	*ABCG2*	25.91	26.31	24.74	25.74	25.84
13	*BMI1*	22.08	22.38	22.00	22.00	22.12
14	*DKK1*	19.88	20.90	19.35	19.80	19.78
15	*ETFA*	21.09	21.39	21.00	21.01	21.14
16	*HDAC1*	22.17	22.21	21.72	22.16	22.23
17	*JAG1*	22.41	22.91	22.54	22.37	22.45
18	*LIN28B*	33.48	38.36	37.13	33.59	33.71
19	*NFKB1*	24.04	25.28	24.08	24.05	24.12
20	*PTCH1*	27.46	27.51	27.78	27.28	27.46
21	*STAT3*	21.64	21.88	21.70	21.50	21.70
22	*WWC1*	24.35	23.35	24.61	24.27	24.38
23	*ACTB*	16.80	16.96	16.37	16.79	16.83
24	*BMP7*	36.21	36.12	38.24	36.77	35.44
25	*DLL1*	27.53	29.09	31.13	27.23	27.62
26	*FGFR2*	28.81	31.79	31.62	31.93	31.88
27	*ID1*	21.52	22.25	22.59	21.41	21.42
28	*JAK2*	25.31	26.11	25.80	25.23	25.26
29	*MAML1*	24.80	24.66	24.78	24.69	24.81
30	*NOS2*	37.64	37.47	38.17	36.85	38.24
31	*PTPRC*	28.31	28.97	34.11	28.30	28.33
32	*YAP1*	21.73	21.96	21.85	21.59	21.74
33	*ALCAM*	20.37	20.37	20.20	20.28	20.38
34	*CD34*	32.79	35.86	36.57	33.10	32.96
35	*DLL4*	30.23	30.93	31.58	29.84	30.12
36	*FLOT2*	23.45	23.44	23.19	23.34	23.47
37	*IKBKB*	24.14	24.48	24.46	23.99	24.06
38	*KIT*	37.23	35.14	37.68	36.35	34.79
39	*MERTK*	24.27	24.23	24.21	24.04	24.12
40	*NOTCH1*	24.05	28.64	29.32	28.49	28.46
41	*RPLP0*	17.81	18.32	18.13	17.68	17.84
42	*TGFBR1*	23.30	23.72	24.16	23.18	23.35
43	*ZEB1*	22.22	25.04	25.86	22.14	22.21
44	*ALDH1A1*	24.45	30.99	30.37	25.76	27.07
45	*CD38*	28.98	32.59	29.47	28.85	28.87
46	*DNMT1*	21.26	21.30	21.09	21.16	21.21
47	*FOXA2*	26.94	27.16	27.85	26.38	27.84
48	*IL8*	21.05	20.47	21.19	21.02	21.02
49	*KITLG*	26.38	26.49	25.44	26.22	26.25
50	*MS4A1*	31.09	32.11	32.48	29.56	29.93
51	*NOTCH2*	23.54	25.98	25.89	25.58	25.81
52	*SAV1*	22.40	22.28	22.21	22.28	22.41
53	*THY1*	27.38	30.88	34.76	27.33	27.39
54	*ZEB2*	26.23	27.58	27.20	25.88	26.11
55	*ATM*	23.65	23.50	23.69	23.56	23.68
56	*CD44*	20.84	23.58	23.52	23.36	23.46
57	*EGF*	26.85	27.15	27.32	26.57	26.62
58	*FOXP1*	21.41	21.64	21.38	21.36	21.38
59	*ITGA2*	20.29	20.57	19.92	20.24	20.30
60	*KLF17*	31.62	31.79	32.08	31.37	31.41
61	*MUC1*	26.75	27.28	28.01	26.53	26.63
62	*PLAT*	22.64	22.37	22.35	22.62	22.56
63	*SIRT1*	24.08	24.05	23.70	24.02	24.52
64	*TWIST1*	29.62	35.70	39.30	29.43	29.40
65	*TBP*	23.95	24.03	23.98	23.80	23.81
66	*ATXN1*	24.81	24.80	25.61	24.70	24.74
67	*CHEK1*	22.57	22.17	22.02	22.07	22.19
68	*ENG*	22.94	29.06	28.82	29.83	30.02
69	*FZD7*	24.05	23.82	23.40	23.94	24.06
70	*ITGA4*	35.02	35.16	36.35	35.33	34.70
71	*KLF4*	29.79	30.18	29.54	29.80	29.67
72	*MYC*	21.16	20.78	20.45	21.12	21.20
73	*PLAUR*	20.55	20.77	20.28	20.42	20.47
74	*SMO*	28.11	30.92	35.08	28.00	28.07
75	*TWIST2*	37.73	Not detected	Not detected	39.43	39.25
76	*GAPDH*	16.02	16.18	15.88	15.96	16.04
77	*AXL*	20.19	20.17	20.02	20.16	20.24
78	*DACH1*	26.65	27.17	26.64	26.85	26.69
79	*EPCAM*	25.25	27.57	27.63	25.27	25.34
80	*GATA3*	27.53	27.45	27.68	27.41	27.50
81	*ITGA6*	20.44	20.63	20.51	20.30	20.41
82	*LATS1*	24.00	23.79	23.71	23.83	24.05
83	*MYCN*	29.94	30.57	34.20	30.19	30.20
84	*POU5F1*	26.06	26.38	30.23	25.64	25.80
85	*SNAI1*	28.02	28.92	29.89	27.81	27.85
86	*WEE1*	23.46	23.69	24.49	23.14	23.24
87	*HPRT1*	22.29	22.79	22.03	21.68	21.99

Supplementary figures

Supplementary Figure S1

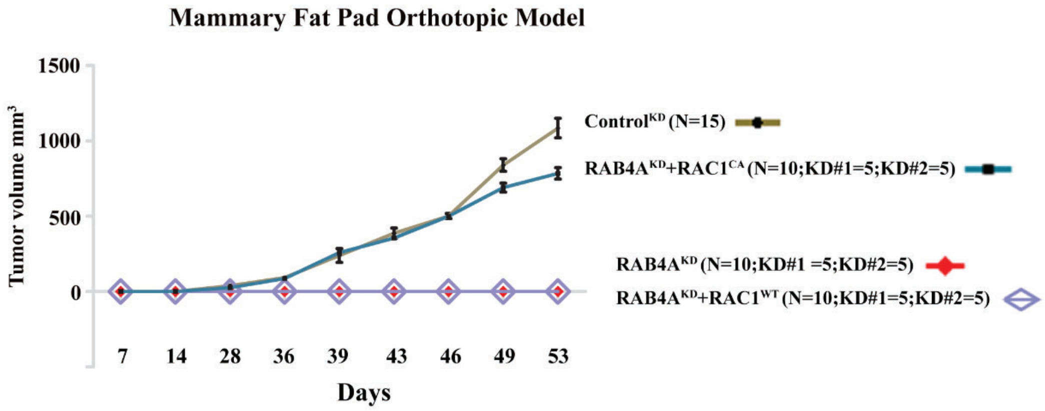

Figure S1. Mammary fat pad orthotopic mouse models that demonstrates the roles of RAB4A and RAC1 regulation of tumor formation. Tumors are derived from sub-cell lines of MDA-MB-231 cells that express either control shRNA or those target RAB4A with and without concurrent $RAC1^{wt}$ or $RAC1^{CA}$ overexpression. The tumor formation and growth are followed over time. Prism software was used for the analysis. The group identities and numbers of mice in each group are written in the inset of the figure [29].

Supplementary Figure S2

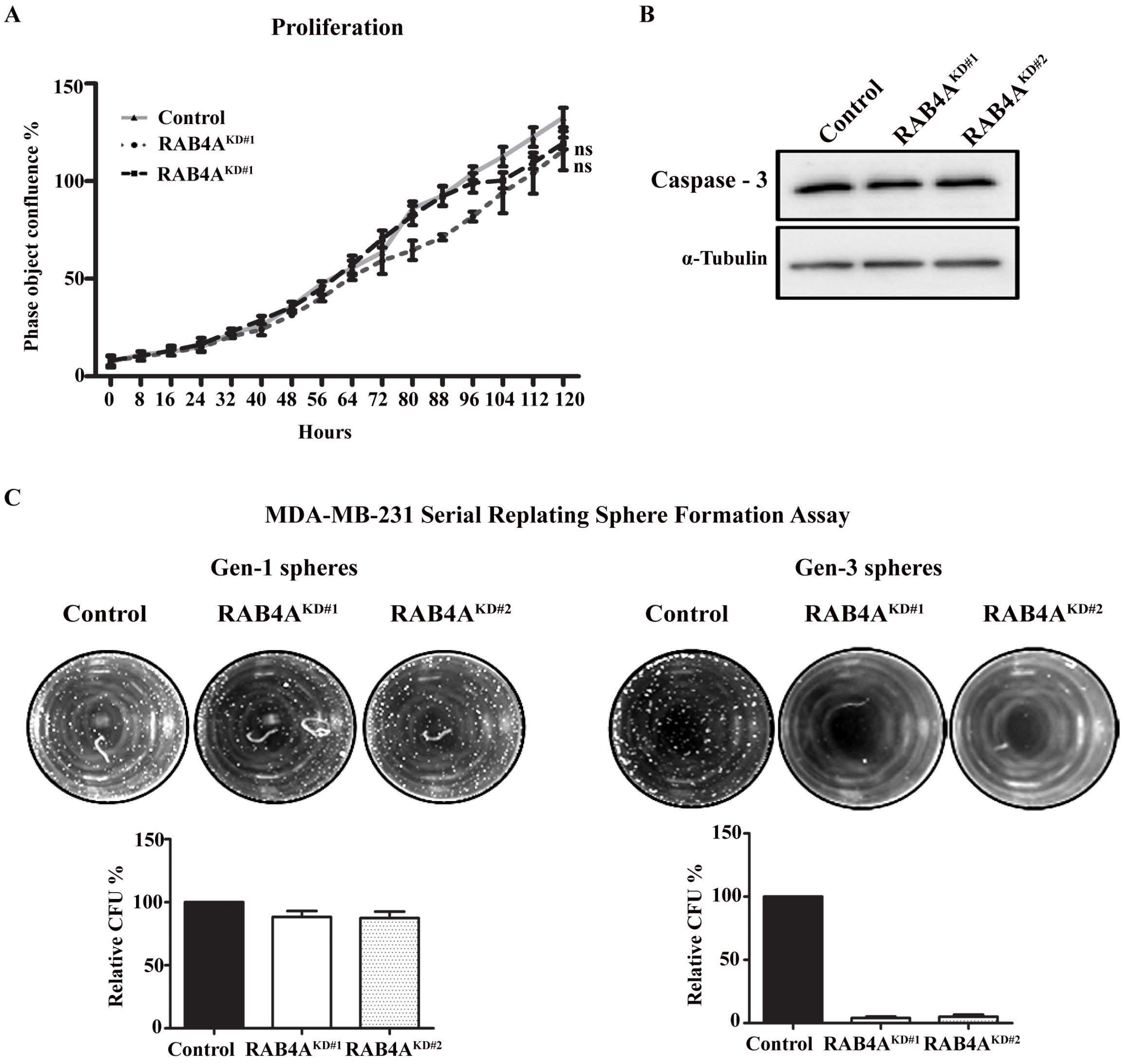

Figure S2. Cell viability assay, immunoblot of Caspase-3, first and third generation of sphere counts from serial-replating sphere formation assay. (A) Viable cell count under adherent culture condition of MDA-MB-231 cells that stably express control shRNA or two distinct shRNAs targeting RAB4A. The numbers of cells were monitored every 8 hours for 5 days employing incuCyte cell imaging machine. (B) Immunoblot quantification of the un-cleaved and cleaved Caspase-3 protein in control cells and RAB4A knockdown cells. (C) Representative microscopic images of the first generation spheres and quantification (left top and bottom), and the same for the third generation spheres (right top and bottom). The analysis was done using Open CFU and Prism; the data presented is from three biological repeats.

Supplementary Figure S3

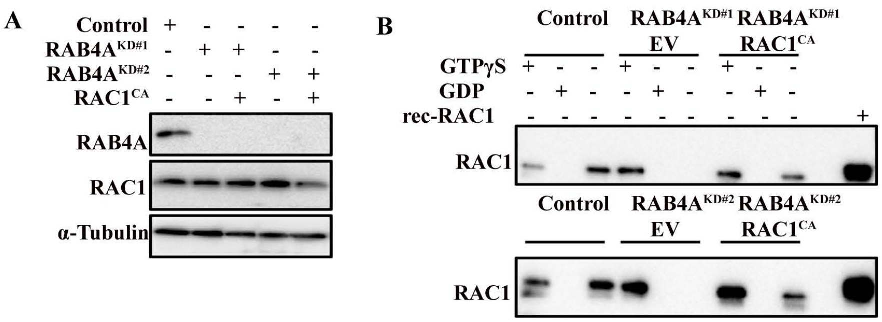

Figure S3. Immunoblot validation of RAB4A knockdown and exogenous RAC1-CA expression. (A) Immunoblot of RAB4A and RAC1. (B) Pull-down of GTP-bound RAC1 to study the activation status in control, RAB4A knockdown alone and concurrent RAB4A KD and RAC1-CA expressing cells. The input for the pull-down is the as shown in (A).

Supplementary Figure S4

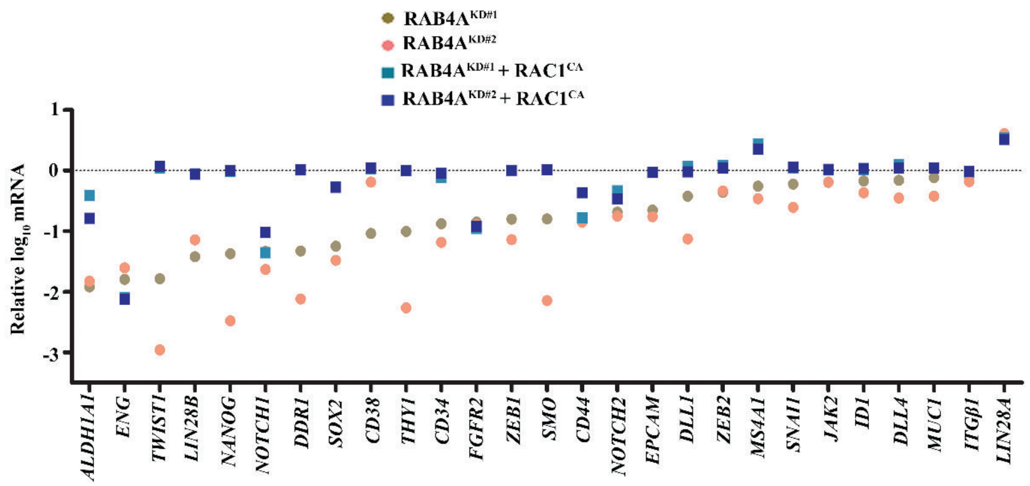

Figure S4. RAB4A controls the transcription of genes that are functionally involved in cancer stemness. This graph shows the expression of the genes that are altered in response to RAB4A knockdown and rescued by constitutively active RAC1. Relative log_{10} mRNA expression was calculated in comparison to the control. The raw expression data is shown in Table S1 above. The values were sorted using RAB4A shRNA from the smallest to the largest.

Supplementary Figure S5

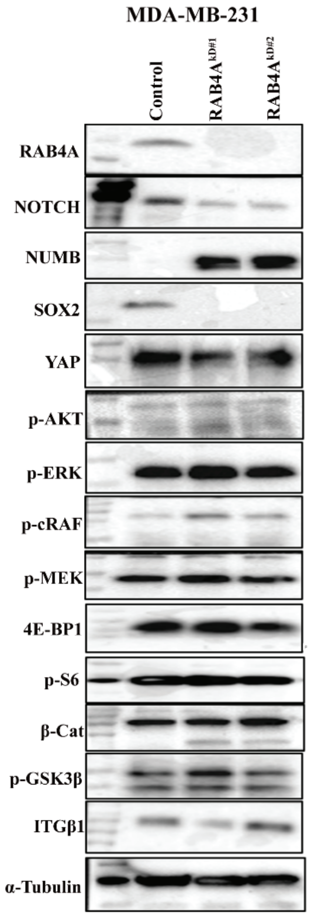

Figure S5. Immunoblots of key cancer pathway proteins. The cell lysates were prepared from MDA-MB-231 stable cell lines containing either the control vector or one of the two RAB4A shRNA expressing vectors.

Supplementary Figure S6

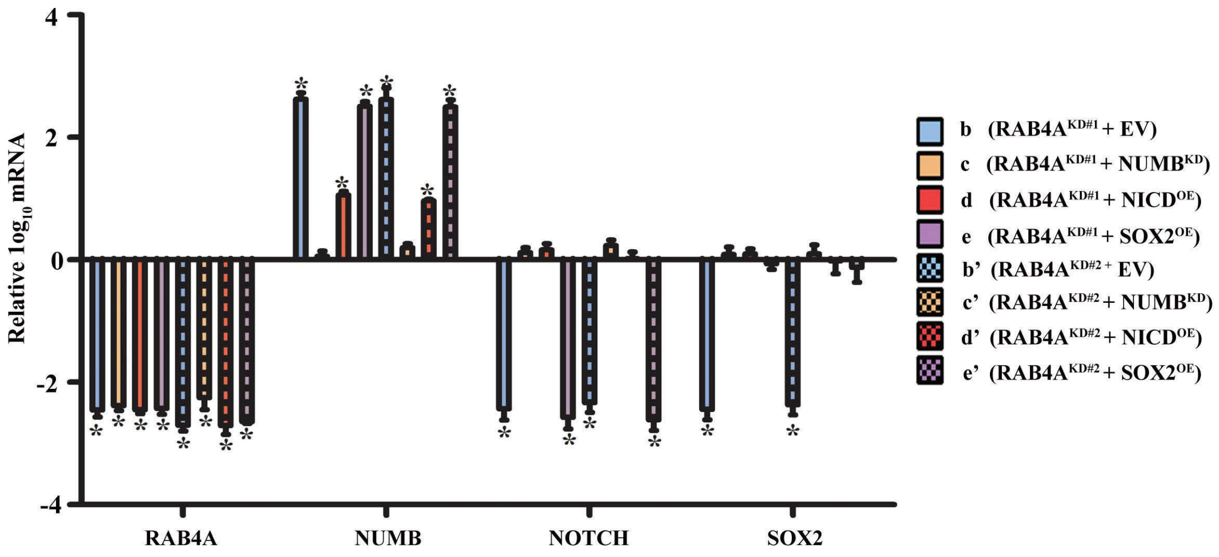

Figure S6. PCR validation of the identities of the cells used for in vivo tumor formation study. The color keys show the presumed genetic identity of each MDA-MB-231 derived stable cell line with the down- and up-regulation of relevant genes. The gene expression for all the genes in the control cells is set at the baseline. "*" represents significant difference between the baseline as set by the control cells and the target cell line labeled by the color keys

Supplementary Figure S7

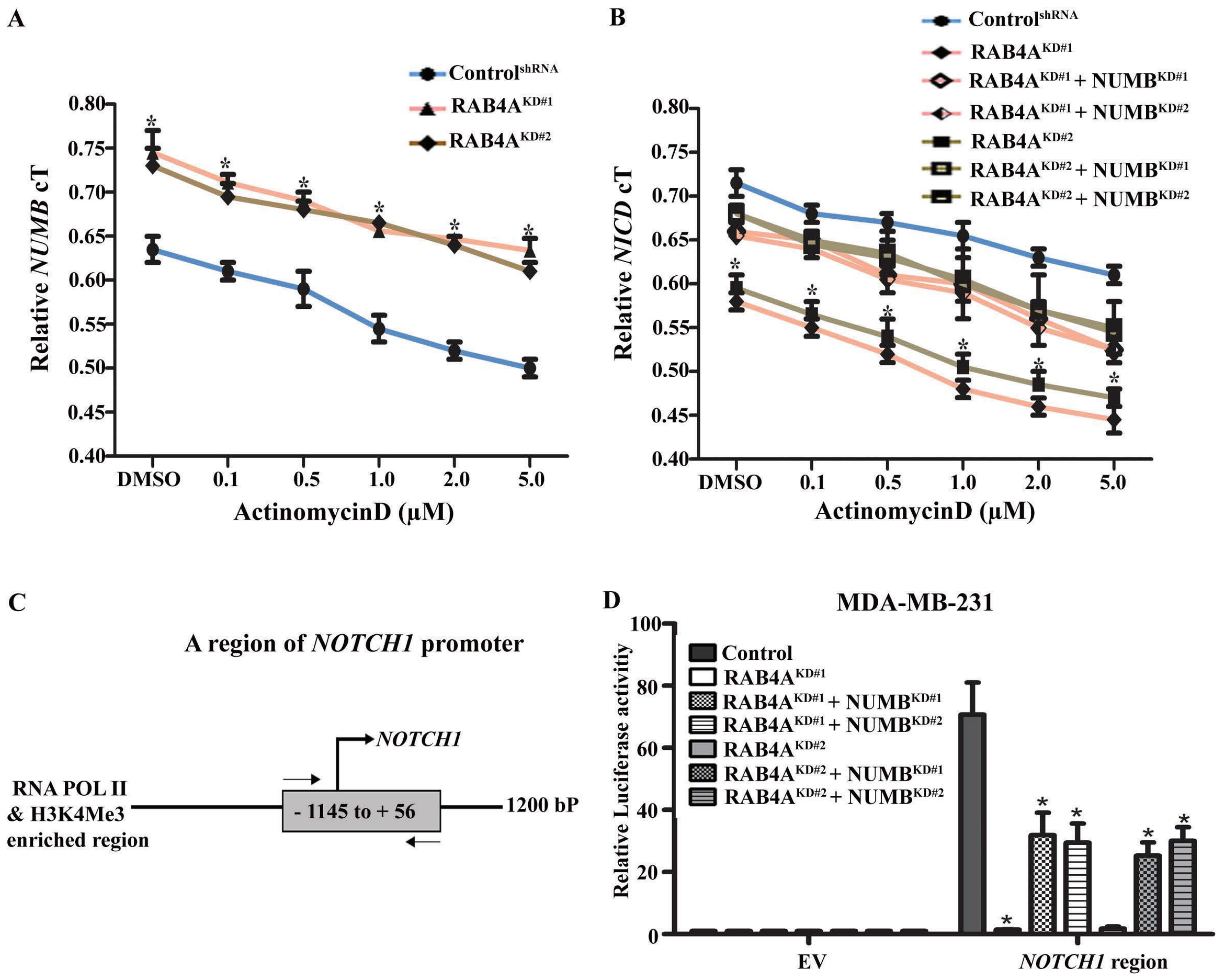

Figure S7. RAB4A negatively regulates NUMB level at the transcription level, as does NUMB regulate NOTCH expression. (A, B) Actinomycin D treatment to study the baseline expression and the rate of transcript reduction induced by Actinomycin D dose-escalation of NUMB (A) and NOTCH1 (B). (C) Schematic representation of NOTCH1 promoter region used for luciferase reporter assay. (D) Luciferase activity assay. The experiment was biologically repeated with similar results. "*" marks the level that is significantly different from that in the control cells.

Supplementary Figure S8

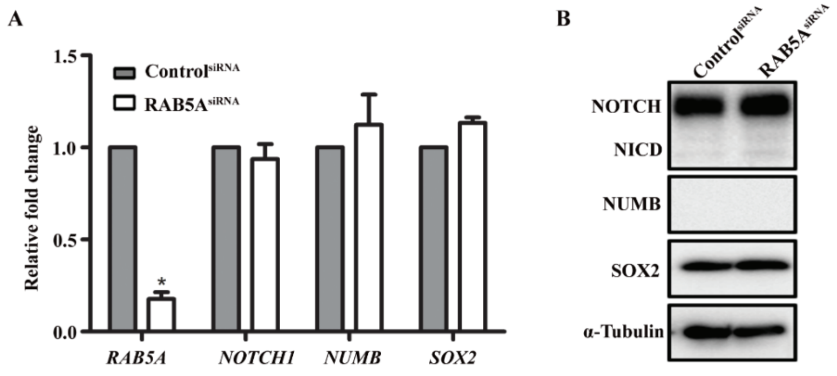

Figure S8. Change in RAB5A expression has no impact on NOTCH, NUMB and SOX2 transcript and protein levels in MDA-MB-231 cells. (A) qPCR analysis on cells with or without RAB5A knockdown. (B) Immunoblots for these proteins in cells as described in (A). Data are presented as mean ± SEM ($n \geq 3$) "*" indicates when $p < 0.05$ compared to control.
