# Supplementary material for: RAB4A is a master regulator of cancer cell stemness upstream of NUMB–NOTCH signaling
Source: Cell Death Dis. 2024 Oct 27;15(10):778. doi: 10.1038/s41419-024-07172-w (PMC11514220; doi:10.1038/s41419-024-07172-w)

Figure 2

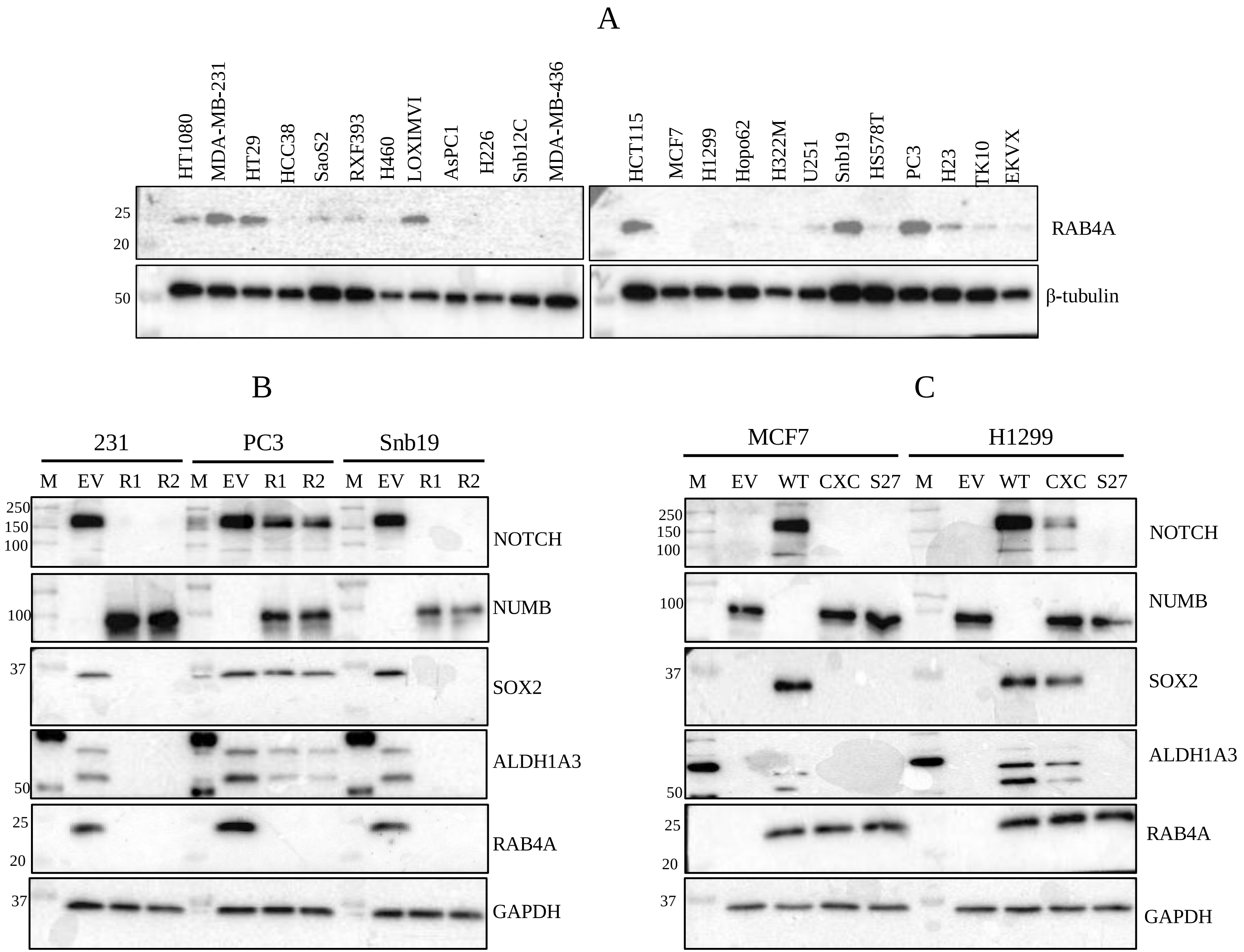
A
HT1080
MDA-MB-231
HT29
HCC38
SaoS2
RXF393
H460
LOXIMVI
AsPC1
H226
Snb12C
MDA-MB-436
HCT115
MCF7
H1299
Hopo62
H322M
U251
Snb19
HS578T
PC3
H23
TK10
EKVX
25
20
50
RAB4A
β-tubulin
B
231
PC3
Snb19
M EV R1 R2 M EV R1 R2 M EV R1 R2
250
150
100
NOTCH
100
NUMB
37
SOX2
ALDH1A3
50
25
20
RAB4A
37
GAPDH
C
MCF7
H1299
M EV WT CXC S27 M EV WT CXC S27
250
150
100
NOTCH
100
NUMB
37
SOX2
ALDH1A3
50
25
20
RAB4A
37
GAPDH

Figure 3

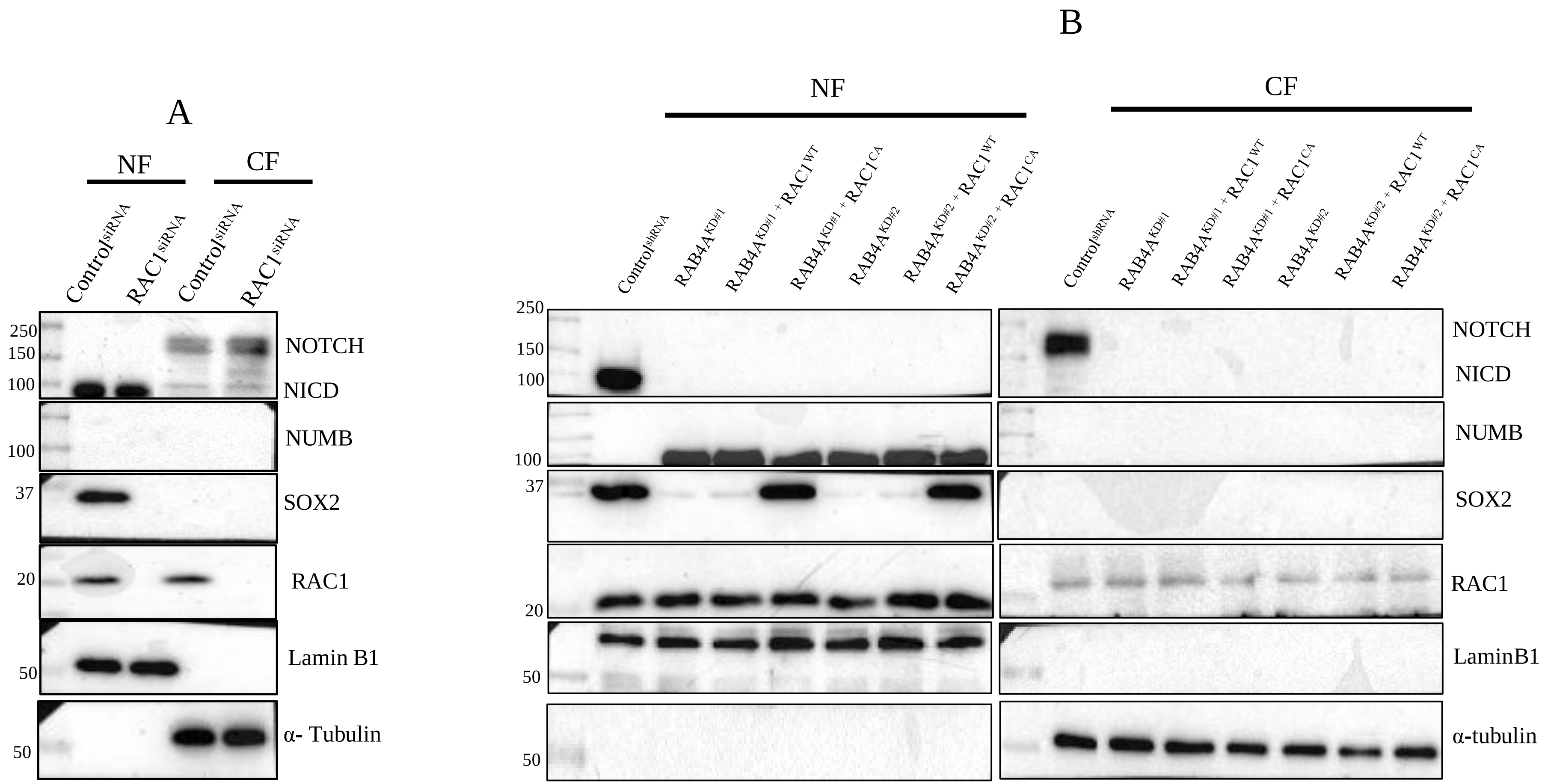
A
NF
CF
Control siRNA
RAC1 siRNA
Control siRNA
RAC1 siRNA
250
150
100
NOTCH
NICD
100
NUMB
37
SOX2
20
RAC1
50
Lamin B1
50
α- Tubulin
B
NF
CF
Control shRNA
RAB4A KD#1
RAB4A KD#1 + RAC1 WT
RAB4A KD#1 + RAC1 CA
RAB4A KD#2
RAB4A KD#2 + RAC1 WT
RAB4A KD#2 + RAC1 CA
250
150
100
NOTCH
NICD
100
NUMB
37
SOX2
20
RAC1
50
LaminB1
50
α-tubulin

Figure 3

C

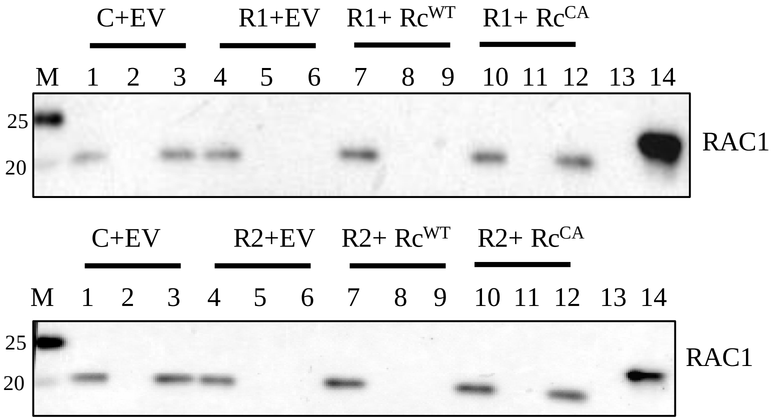

Lane 1, 4, 7 and 10 – Lysate + GTP γS
Lane 2, 5, 8 and 11 – Lysate + GDP
Lane 3, 6, 9 and 12 – Lysate
Lane 13 - Empty
Lane 14 - Recombinant RAC1 control

Input

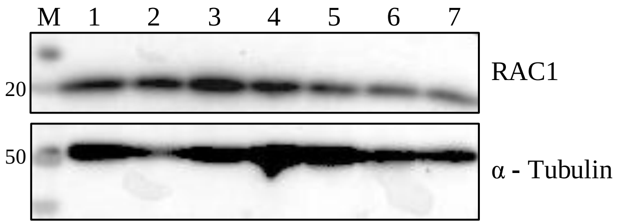

Lane 1 – C + EV
Lane 2 – R1 + EV
Lane 3 – R1 + RAC1-WT
Lane 4 – R1 + RAC1-CA
Lane 5 – R2 + EV
Lane 6 – R2 + RAC1-WT
Lane 7 – R2 + RAC1-CA

D

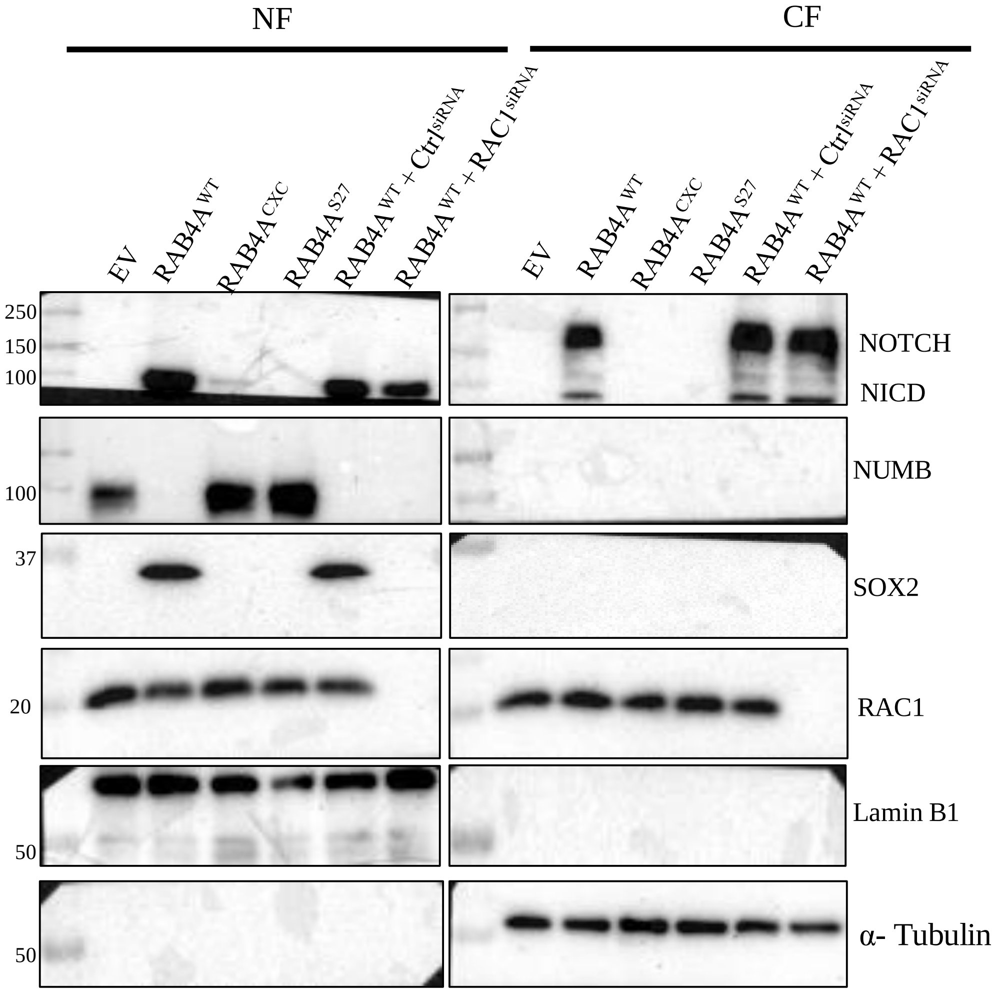

Figure 3

E

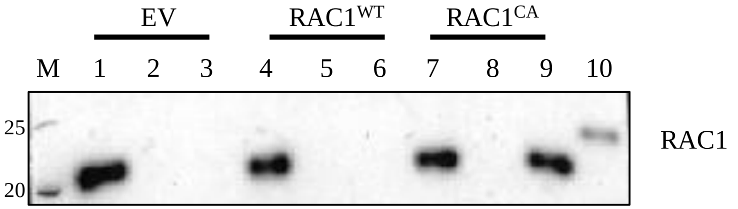

Lane 1, 4 and 7 – Lysate + GTP γS
Lane 2, 5 and 8 – Lysate + GDP
Lane 3, 6 and 9 – Lysate
Lane 10 - Recombinant RAC1 control

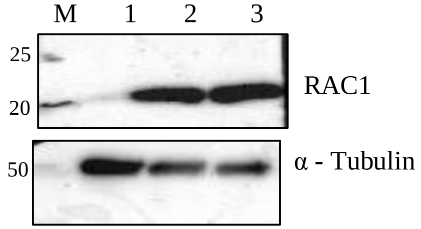

Lane 1 – EV
Lane 2 – RAC1-WT
Lane 3 – RAC1-CA

F

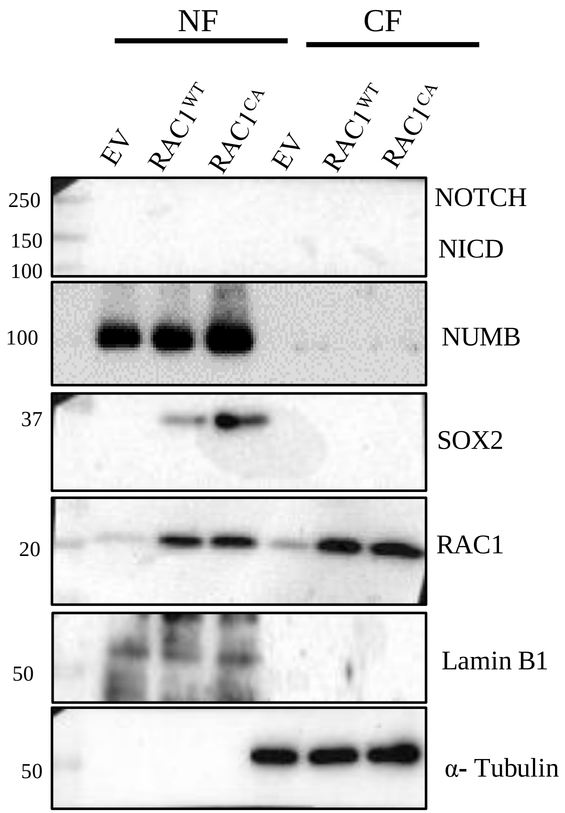

Figure 4

A

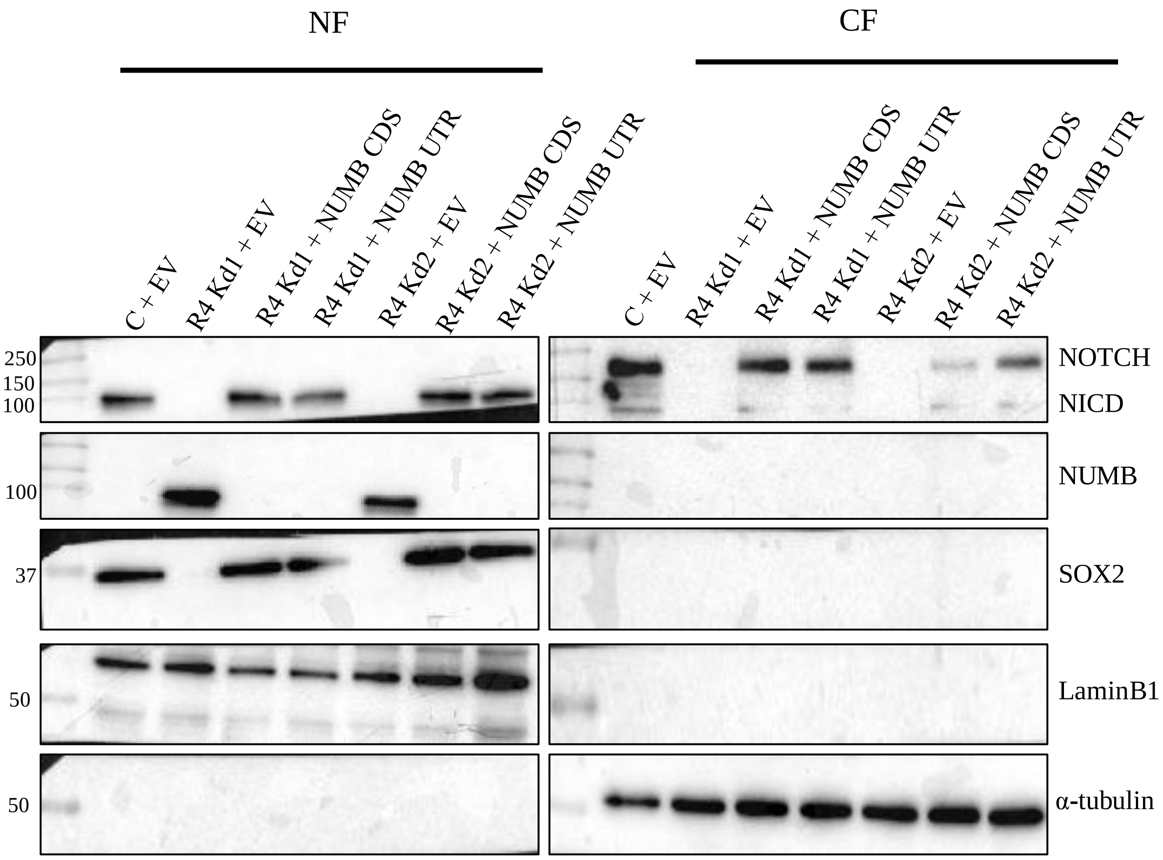

B

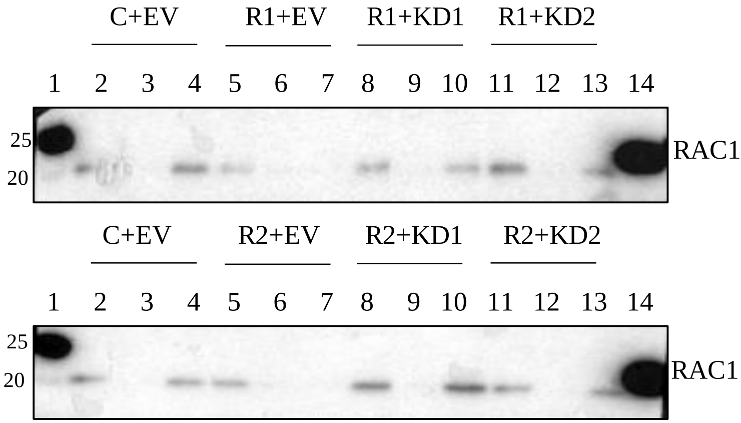

Lane 2, 5, 8 and 11 – Lysate + GTP γS
Lane 3, 6, 9 and 12 – Lysate + GDP
Lane 4, 7, 10 and 13 – Lysate
Lane 14 - Recombinant RAC1 control

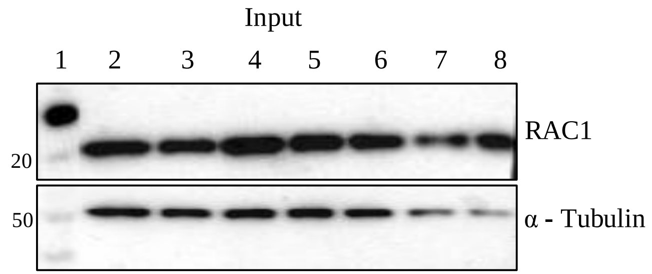

Lane 2 – Control +EV
Lane 3 – R1 +EV
Lane 4 – R1 + NUMB KD#1 CDS
Lane 5 - R1 + NUMB KD#2 UTR
Lane 6 – R2 +EV
Lane 7 – R2 + NUMB KD#1 CDS
Lane 8 – R2 + NUMB KD#2 UTR

Figure 4

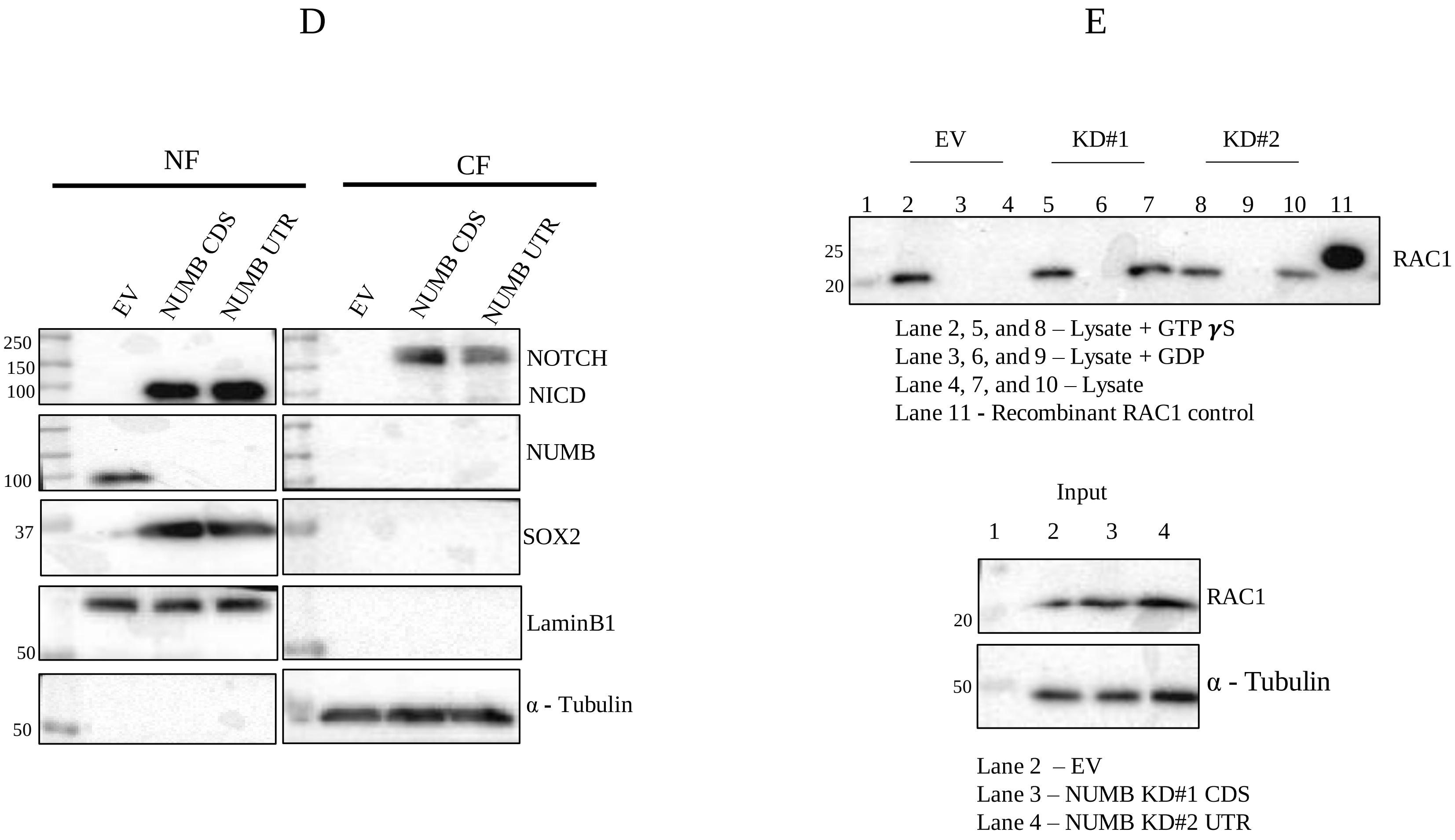
D
NF
CF
EV
NUMB CDS
NUMB UTR
EV
NUMB CDS
NUMB UTR
250
150
100
NOTCH
NICD
100
NUMB
37
SOX2
50
LaminB1
50
α - Tubulin
E
EV
KD#1
KD#2
1 2 3 4 5 6 7 8 9 10 11
25
20
RAC1
Lane 2, 5, and 8 – Lysate + GTP γS
Lane 3, 6, and 9 – Lysate + GDP
Lane 4, 7, and 10 – Lysate
Lane 11 - Recombinant RAC1 control
Input
1 2 3 4
20
RAC1
50
α - Tubulin
Lane 2 – EV
Lane 3 – NUMB KD#1 CDS
Lane 4 – NUMB KD#2 UTR

Figure 5

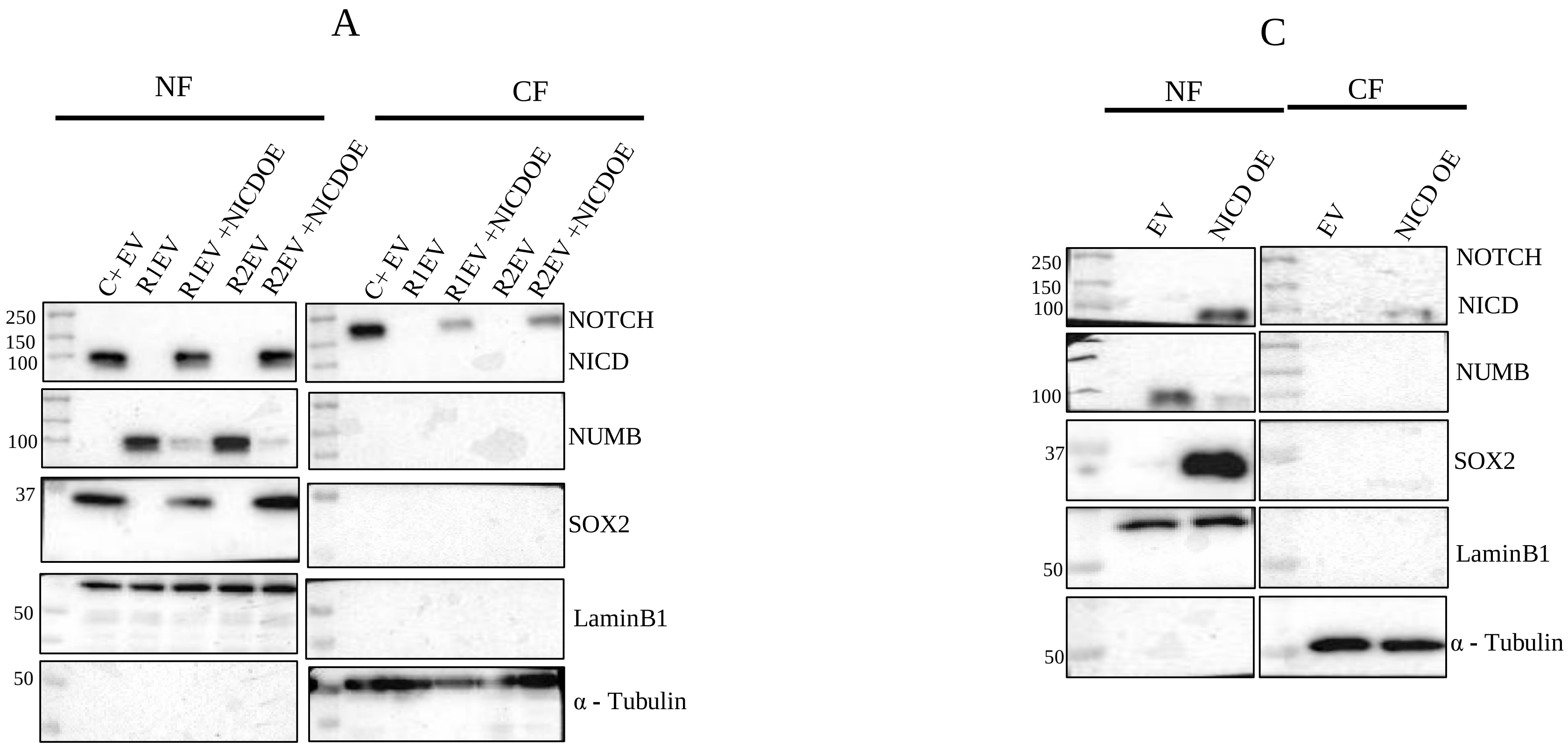
A
NF
CF
C+ EV
R1EV
R1EV +NICDOE
R2EV
R2EV +NICDOE
250
150
100
37
50
NOTCH
NICD
NUMB
SOX2
LaminB1
α - Tubulin
C
EV
NICD OE

Figure 5

E

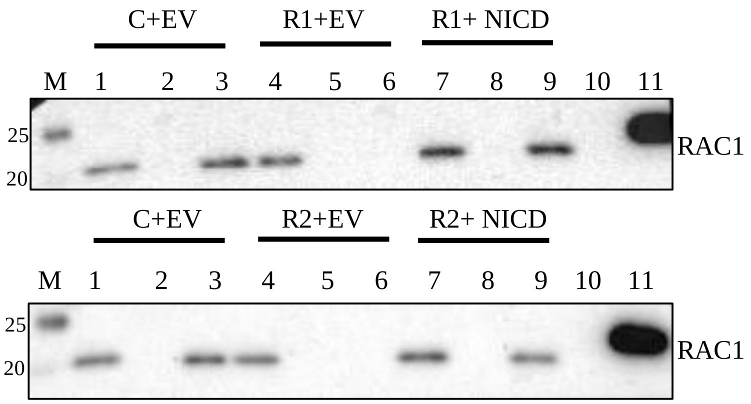

Lane 1, 4, and 7 – Lyaste + GTP γS
Lane 2, 5, and 8 – Lyaste + GDP
Lane 3, 6, and 9 – Lysate
Lane 10 - Empty
Lane 11 - Recombinant RAC1 control

Input

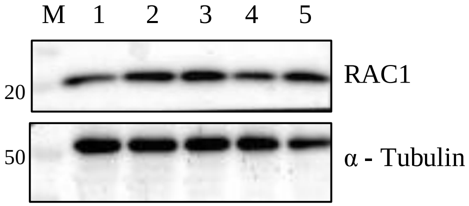

Lane 1 – C+EV
Lane 2 – R1+EV
Lane 3 – R1+NICD
Lane 4 – R2+EV
Lane 5 – R2+ NICD

F

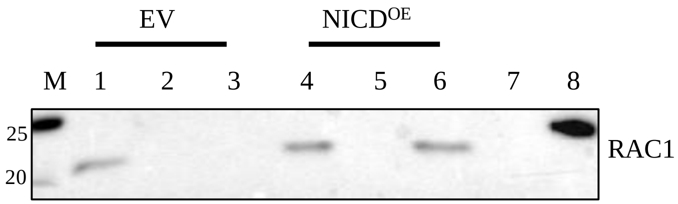

Lane 1, and 4 – Lyaste + GTP γS
Lane 2, and 5 – Lyaste + GDP
Lane 3, and 6 – Lysate
Lane 8 - Recombinant RAC1 control

Input

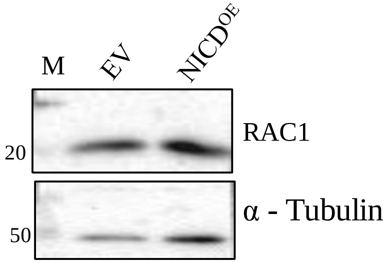

Figure 6

A

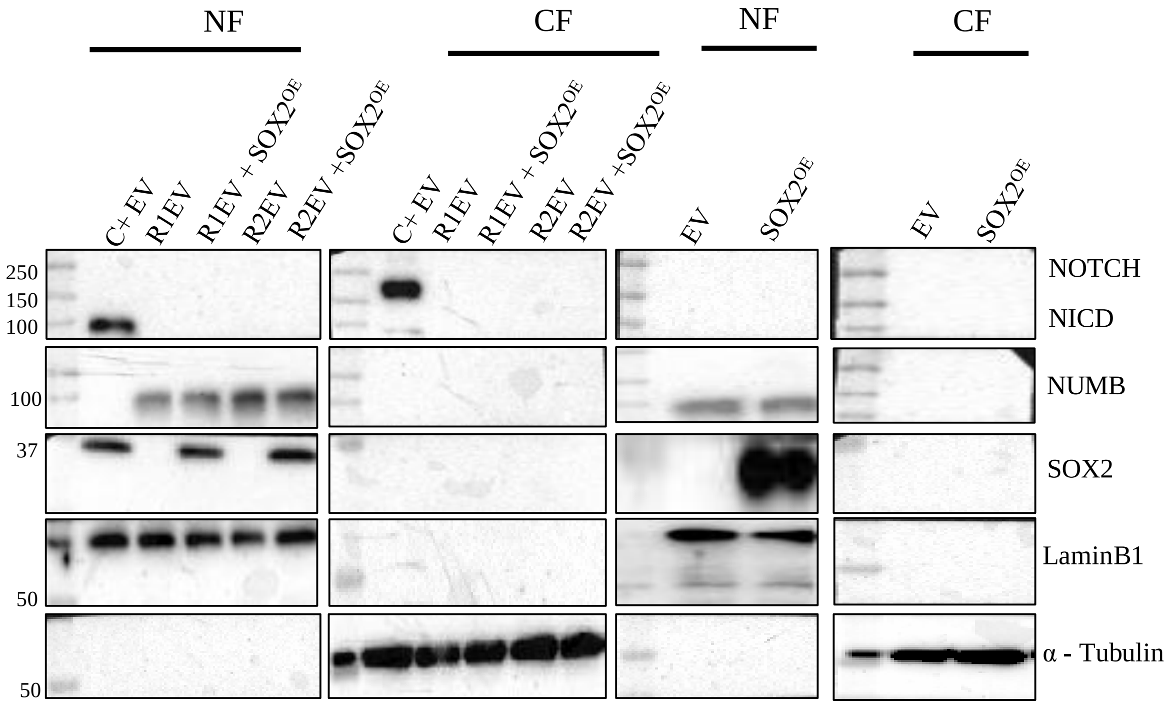

NF
CF
NF
CF
C+ EV
R1EV
R1EV + SOX2OE
R2EV
R2EV +SOX2OE
C+ EV
R1EV
R1EV + SOX2OE
R2EV
R2EV +SOX2OE
EV
SOX2OE
EV
SOX2OE
250
150
100
100
37
50
50
NOTCH
NICD
NUMB
SOX2
LaminB1
α - Tubulin

Figure 6

B

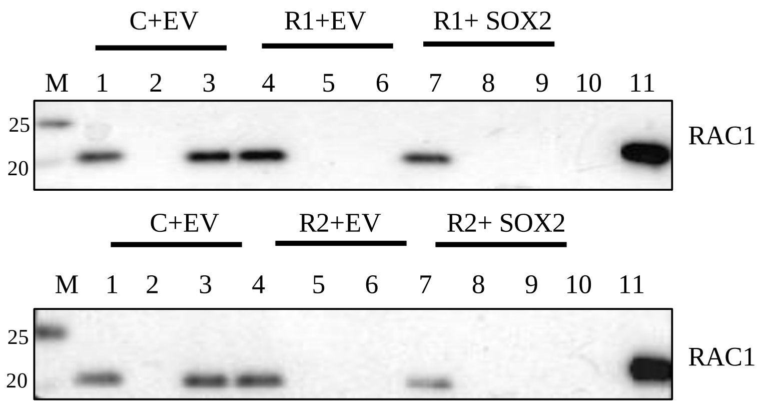

Lane 1, 4, and 7 – Lysate + GTP γS
Lane 2, 5, and 8 – Lysate + GDP
Lane 3, 6, and 9 – Lysate
Lane 10 - Empty
Lane 11 - Recombinant RAC1 control

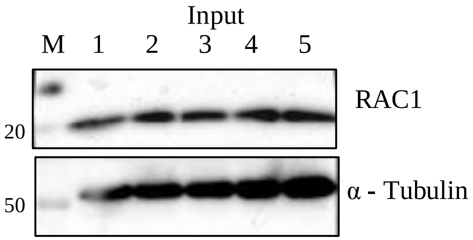

Lane 1 – C+EV
Lane 2 – R1+EV
Lane 3 – R1+SOX2
Lane 4 – R2+EV
Lane 5 – R2+ SOX2

C

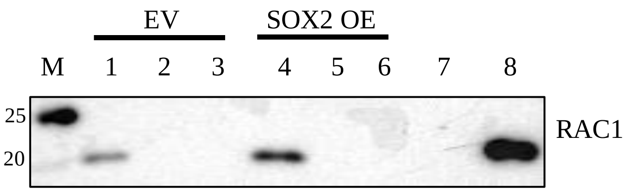

Lane 1 and 4 – Lysate + GTP γS
Lane 2 and 5 – Lysate + GDP
Lane 3 and 6 – Lysate
Lane 7 - Empty
Lane 8 - Recombinant RAC1 control

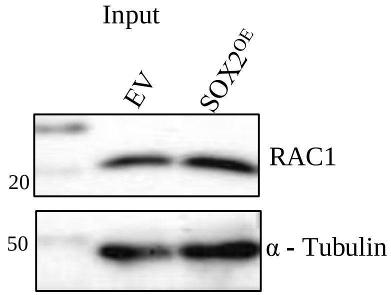

Supplementary Figure S2 B

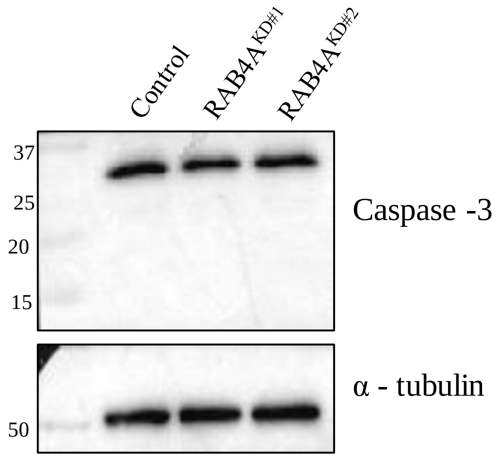

Supplementary Figures S3

A

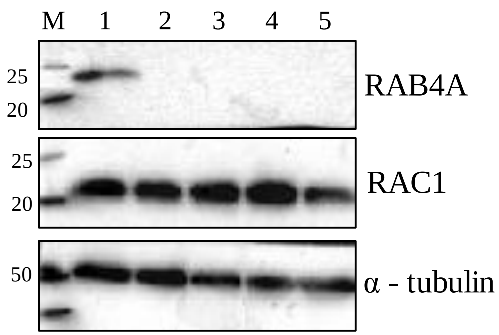

Lane 1 – Control
Lane 2 – R4Kd#1
Lane 3 – R4Kd#1 + $RAC1^{CA}$
Lane 4 – R4Kd#2
Lane 5 – R4Kd#1 + $RAC1^{CA}$

B

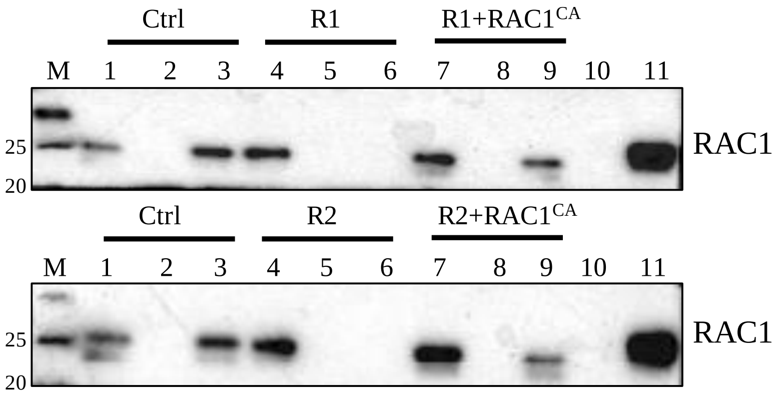

Lane 1, 4 and 7 – Lyaste + GTP γS
Lane 2, 5 and 8 – Lyaste + GDP
Lane 3, 6 and 9 – Lysate
Lane 11 - Recombinant RAC1 control

Supplementary Figure – S5 & S8

S5

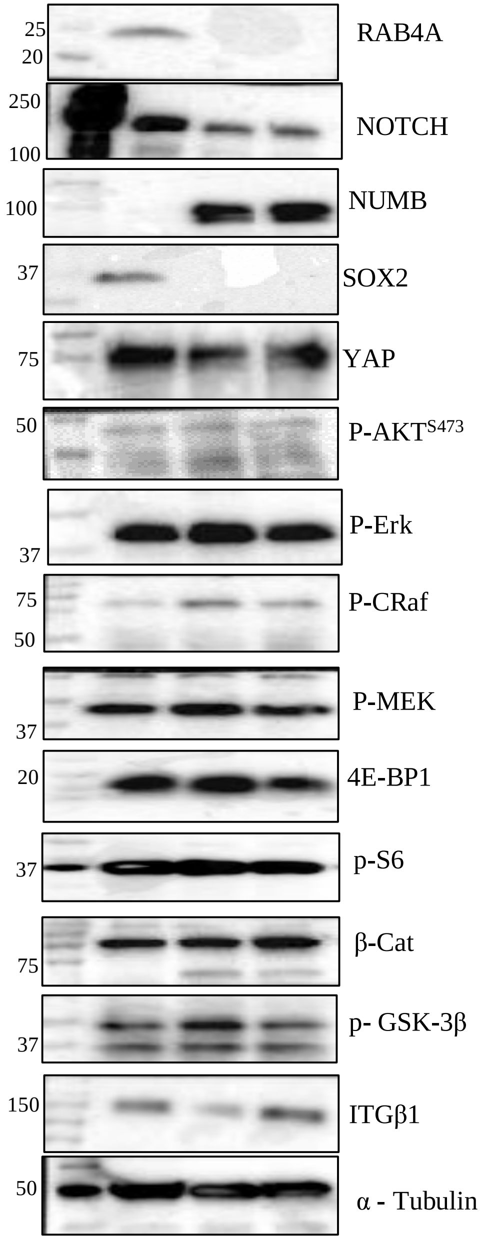

S8

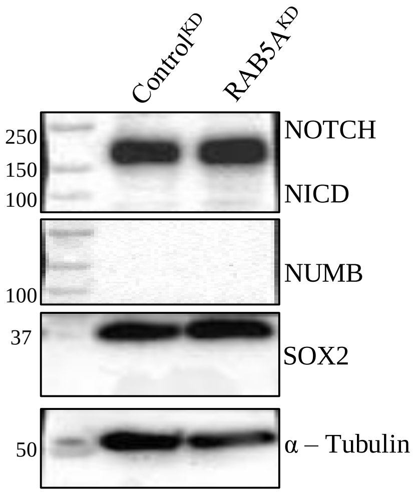

Supplement: Supplementary file 2 — Full Blots [file 41419_2024_7172_MOESM2_ESM.pdf]
